# Cation effects on CO_2_ reduction catalyzed by single-crystal and polycrystalline gold under well-defined mass transport conditions

**DOI:** 10.1126/sciadv.adr6465

**Published:** 2025-02-07

**Authors:** Zhihao Cui, Andrew Jark-Wah Wong, Michael J. Janik, Anne C. Co

**Affiliations:** ^1^ Department of Chemistry and Biochemistry, Ohio State University, Columbus, OH 43210, USA.; ^2^ Department of Chemical Engineering, Pennsylvania State University, University Park, PA 16802, USA.

## Abstract

The presence of alkali metal cations in the electrolyte substantially affects the reactivity and selectivity of electrochemical carbon dioxide (CO_2_) reduction (CO_2_R). This study examines the role of cations in CO_2_R on single-crystal and polycrystalline Au under controlled mass-transport conditions. It establishes that CO_2_ adsorption is the rate-determining step regardless of cation type or surface structure. Density functional theory calculations show that electron transfer occurs to a solvated CO_2_-cation complex. A more positive potential of zero charge enhances CO_2_R activity only on Au with similar surface coordination. The symmetry factor (β) of the rate-determining step varies with surface structure and cation identity, with density functional theory calculations indicating β’s sensitivity to surface and double-layer structures. These findings emphasize the importance of both surface and double-layer structures in understanding cation effects on CO_2_R.

## Teaser

Alkali metal cations’ influence on CO_2_ electroreduction was investigated using Au electrodes with varying surface structures.

## INTRODUCTION

The electrochemical carbon dioxide (CO_2_) reduction (CO_2_R) process driven by renewable electricity is a promising technique to convert relatively inert CO_2_ gas into useful chemicals and fuels (*1*–*3*). Since the pioneering work of Akira and Hori, it is known that alkali metal cations in the electrolyte alter the reaction activity and product selectivity of CO_2_R (*4*). CO_2_R activity has been observed to decrease in the order of Cs^+^ > K^+^ > Na^+^ > Li^+^, with up to two orders of magnitude difference in CO_2_R activity between Li^+^ and Cs^+^ on various coinage metals under identical applied potential (*5*–*7*). Several hypotheses have been proposed for the role of cations, including interfacial pH buffering (*8*, *9*), changes in interfacial electrical field (*5*, *10*), electrostatic interaction with reaction intermediates (*7*, *11*), chemical interaction with adsorbed intermediates through specific adsorption (*12*, *13*), reorganization of interfacial water (*14*, *15*), non–electric field effect (*16*, *17*), and cation-coupled electron transfer (*18*, *19*). However, these effects are often intertwined and challenging to quantify experimentally (*20*, *21*), making a comprehensive molecular-level understanding of alkali metal cation effects on CO_2_R a subject of ongoing debate.

Koper and co-workers reported notable surface structure sensitivity of cations during CO reduction on Cu single-crystal electrodes, where the onset potential for ethylene formation depends on both the cation identity and surface structure on Cu(*hkl*) (*22*). Other studies on CO_2_R to CO on Au have predominantly focused on polycrystalline surfaces. Systematic investigations using well-defined single-crystal electrodes could help distinguish cation effects from surface structure effects in CO_2_R. In addition to cation interactions, the activity and selectivity of CO_2_R on different catalysts also depend on the mass transport conditions (*23*, *24*). For example, Goyal *et al.* demonstrated that the faradic efficiency of CO_2_ to CO on Au increased by nearly 20% with improved mass transport (*25*).

Here, experiments were conducted to meticulously control the surface structure, mass transport conditions, and electrolyte solution purity to investigate how cations influence the catalysis of CO_2_R on Au surfaces. Three single-crystal Au electrodes, Au(100), Au(110), and Au(111), along with two polycrystalline Au (pcAu) electrodes prepared using different surface cleaning methods [electrochemically polished Au (EP-pcAu) and flame-annealed Au (FA-pcAu)], were studied in CO_2_-saturated LiHCO_3_, NaHCO_3_, KHCO_3_, RbHCO_3_, and CsHCO_3_ electrolytes using a rotating ring-disk electrode (RRDE) assembly. The Au ring electrode served as a real-time detector for quantifying CO generated from the Au disk (*25*, *26*). The study revealed that alkali metal cations exhibit surface structure–dependent effects. For pcAu, the observed trends with cations varied substantially depending on the surface preparation methods (e.g., flame annealed versus electrochemically polished). Overall, the promotional effects of cations vary across different single-crystal and polycrystalline surface structures. Furthermore, cations were found to influence the potential of zero charge (PZC) values, and a positive correlation between the CO_2_R activity and PZC was observed only on Au surfaces with comparable surface coordination numbers.

Combining Tafel analysis and kinetic isotope effect (KIE) experiments, this study demonstrates that the rate-determining step (RDS) is a CO_2_ adsorption step coupled with electron transfer, irrespective of the cation identity and surface structure. The symmetry factor (β) of the RDS was found to vary with the cation identity and the surface structure, indicating that cations play a crucial role in the electrokinetics of CO_2_R. Density functional theory (DFT) calculations of the symmetry factors suggest that electron transfer occurs to a solvated CO_2_-cation complex, providing insights into the cation-dependent electrokinetics observed during CO_2_R.

## RESULTS

### Electrochemical characterization of the Au catalyst surface

**Fig. 1.**
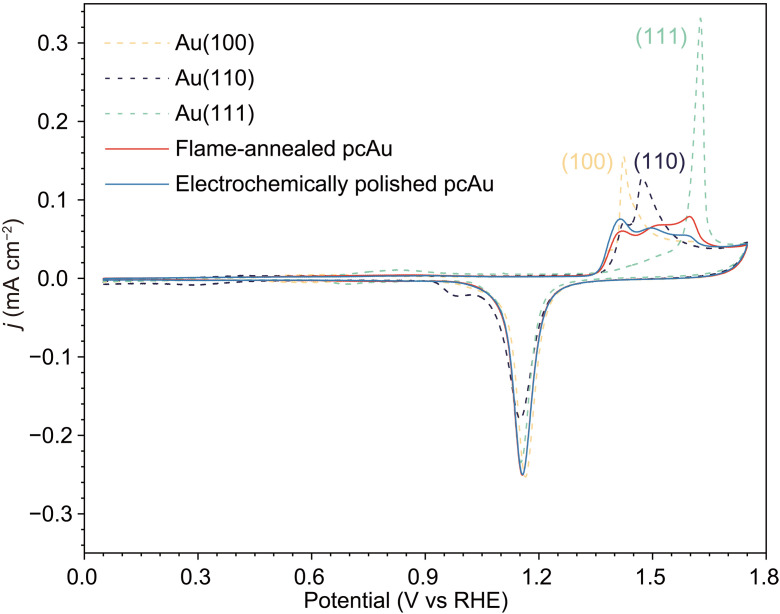
Cyclic voltammetry characterization of different Au electrodes. CVs of Au(100), Au(110), Au(111) single-crystal pcAu, EP-pcAu, and FA-pcAu recorded at 50 mV s^−1^ in Ar-saturated 0.1 M H_2_SO_4_ with a rotation rate of 1600 rpm.

Cation dependence on CO_2_R reaction was obtained on well-characterized single-crystal and pcAu electrodes. To verify the distribution of surface facet on each Au electrode, we recorded cyclic voltammograms (CVs) of Au oxidation immediately after CO_2_R experiments (Fig. 1). Typical voltammetric features such as a broad double-layer region (from 0 to 1.3 V_RHE_) and distinct gold oxide peaks serve as characteristic fingerprints of a clean Au(*hkl*)/electrolyte interface (*27*). Additional voltammograms, depicted in fig. S1, confirm that thermally induced surface reconstruction of Au (*28*) persists during active CO_2_ electrolysis, evidenced by discernible lifting peaks within the double-layer region even after the CO_2_R experiment. For simplicity, reconstructed Au surfaces like Au(100)-hex, Au(110)-(1 × 2), and Au(111)-(√3 × 22) are abbreviated as Au(100), Au(110), and Au(111), respectively, with their corresponding surface coordination numbers (CNs) of 9, 7, and 9, respectively. The correlation between Au oxide peaks and their surface structures is well documented on pcAu electrodes (*29*, *30*), where peak potentials for Au(100), Au(110), and Au(111) planes are ~1.42, 1.45, and 1.58 V_RHE_ (*31*), respectively. These Au oxidation peaks are used here to qualitatively infer the distribution of surface facets on pcAu.

The same pcAu electrode exhibits varying surface facet distributions depending on the surface treatment method. Electrochemical polishing (EP-pcAu) commonly exposes predominantly Au(100)-like surface features, while flame annealing (FA-pcAu) results in mainly Au(111) facets (*30*). These voltammetric features offer a straightforward qualitative comparison between pcAu surfaces, although quantifying the exact facet distribution is complex. The surface facet distribution of the pcAu electrode appears consistent before and after CO_2_R, as depicted in fig. S2. Subsequent sections of this study report that pcAu electrodes prepared with different surface treatments exhibit distinct intrinsic CO_2_R activities and respond differently to cation effects.

### Cation dependence of CO_2_R

**Fig. 2.**
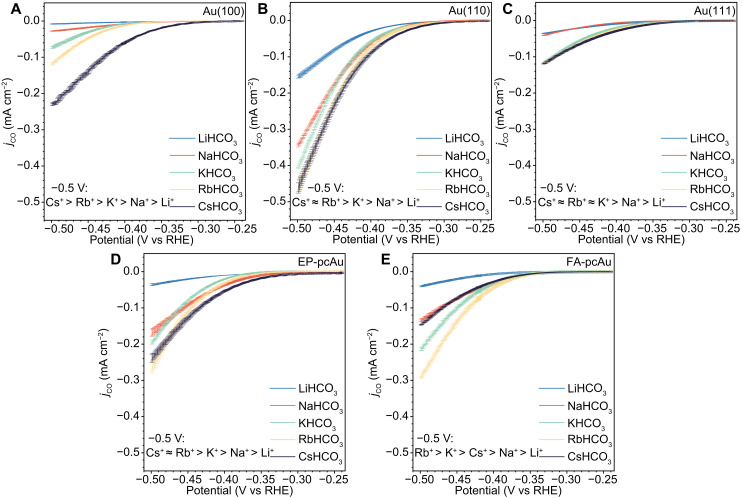
Partial current densities of CO measured by RRDE voltammetry on different Au electrodes in 0.1 M alkali metal bicarbonate electrolytes. Partial current densities of CO measured on (**A**) Au(100), (**B**) Au(110), (**C**) Au(111), (**D**) EP-pcAu, and (**E**) FA-pcAu during CO_2_R at 15 mV s^−1^ and 1600 rpm in CO_2_-saturated 0.1 M bicarbonate electrolytes (pH = 6.8, *T* = 25 ± 1°C). The current densities represent the average values, and the error bars (SDs) are calculated on the basis of three independent experiments.

The partial current densities of CO generated during CO_2_R on various Au electrodes in 0.1 M MHCO_3_ (M = Li, Na, K, Rb, and Cs) electrolytes are illustrated in Fig. 2. Across different Au catalysts, no consistent cation trend is observed when comparing CO_2_R activity at the same applied potential. Instead, the promotional effect of cations varies depending on the surface structure and the potential window. For instance, on Au(100), CO_2_R activity decreases in the order of Cs^+^ > Rb^+^ > K^+^ > Na^+^ > Li^+^ at −0.5 V_RHE_ (Fig. 2A). Conversely, on Au(111), relative CO_2_R activity shows Cs^+^ ≈ Rb^+^ ≈ K^+^ > Na^+^ > Li^+^ (Fig. 2C), where Cs^+^, Rb^+^, and K^+^ are statistically similar. A FA-pcAu catalyst, rich in Au(111) features, exhibits a different cation trend with Rb^+^ > K^+^ > Cs^+^ > Na^+^ > Li^+^ (Fig. 2E) at −0.5 V_RHE_. This unexpected variation suggests that the pcAu surface cannot be simply considered as a linear combination of the contributions from Au(100), Au(110), and Au(111) surfaces. High-index surface facets, defect sites, and grain boundaries likely play crucial roles in shaping the observed cation trends on pcAu surfaces. These findings indicate that the impact of cations on interfacial pH buffering (*5*) or mean electric double-layer field (*32*) cannot solely account for the observed cation effects at this potential. If these factors were dominant, a consistent cation trend (Cs^+^ > Rb^+^ > K^+^ > Na^+^ > Li^+^) would be expected regardless of the electrode surface structure. Therefore, alternative mechanisms must be considered to explain the structure-sensitive cation effects observed.

### Correlating PZC with CO_2_R activity

**Fig. 3.**
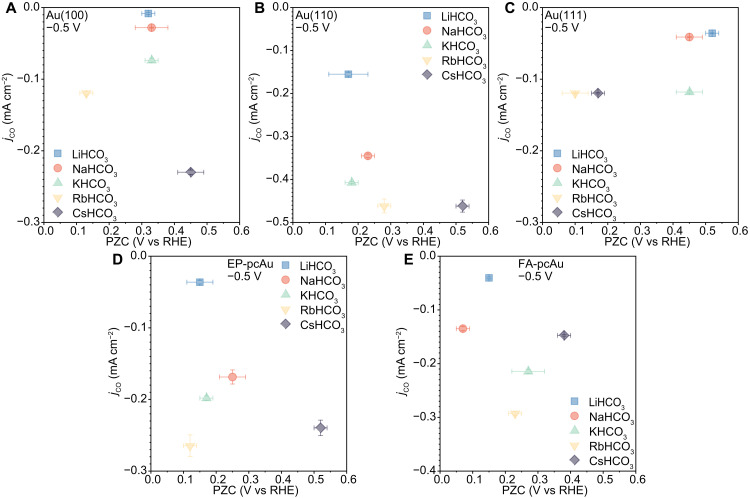
Partial current densities of CO plotted as a function of PZC at −0.5 V_RHE_ on the same Au electrode. Partial current densities of CO measured on (**A**) Au(100), (**B**) Au(110), (**C**) Au(111), (**D**) EP-pcAu, and (**E**) FA-pcAu in CO_2_-saturated 0.1 M bicarbonate electrolytes (pH = 6.8, *T* = 25 ± 1°C). Error bars (SDs) are calculated on the basis of three independent experiments.

To explore the potential origins of the observed cation effects, we plotted the partial current densities of CO at −0.5 V_RHE_ (~−0.9 V_SHE_) against PZC, as depicted in Fig. 3. The PZC values were determined according to the methods outlined in Materials and Methods. Previous experimental and multiscale modeling studies (*23*, *33*–*35*) suggest that CO_2_R activity on Au is primarily governed by the reductive adsorption of CO_2_ within a potential range of −0.7 to −1.0 V_SHE_. Our own investigations in this study (see Supplementary Note 1) corroborate this argument, where surface charge density has been proposed as a descriptor for the CO_2_ adsorption step. Specifically, a more positive PZC results in higher negative surface charge density at the same cathodic potential, thereby favoring CO_2_ adsorption (*5*, *34*). According to this hypothesis, if cation effects are correlated with surface charge density, the corresponding variations in the measured PZC values would be observed.

**Fig. 4.**
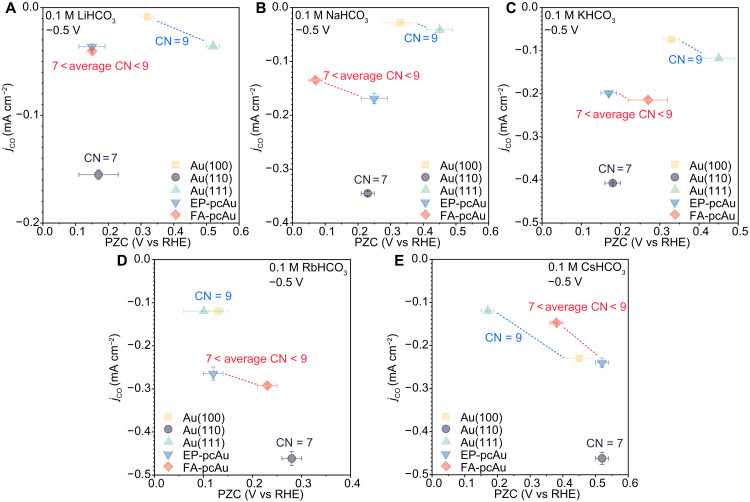
Partial current densities of CO plotted as a function of PZC at −0.5 V_RHE_ in the same electrolyte. Partial current densities of CO measured in CO_2_-saturated 0.1 M (**A**) LiHCO_3_, (**B**) NaHCO_3_, (**C**) KHCO_3_, (**D**) RbHCO_3_, and (**E**) CsHCO_3_ electrolytes (pH = 6.8, *T* = 25 ± 1°C). The Au electrodes with comparable surface CN are linked with dashed lines for a better comparison. Error bars (SDs) are calculated on the basis of three independent experiments.

Cations have been observed to influence the PZC on Au surfaces (fig. S11). However, further investigation reveals that there is no clear correlation between the PZC value and the partial current density of CO (indicative of CO_2_R activity) when varying the cation. For example, on Au(111), similar CO_2_R activities are observed in K^+^-, Rb^+^-, and Cs^+^-containing electrolytes despite a notable 0.35 V difference in PZC between Rb^+^ and K^+^ electrolytes (Fig. 3C). In addition, electrolytes containing Rb^+^ generally exhibit higher CO_2_R activities compared to those with Na^+^ and Li^+^ on the same Au electrode, even though the corresponding PZC values in Rb^+^ can vary widely, being either more positive or negative compared to the other ions. When comparing partial CO current densities as a function of PZC for the same cation across different catalytic surfaces (Fig. 4), Au(100) shows higher CO_2_R activities compared to Au(111) despite having a more positive PZC in the Cs^+^-containing electrolyte. Conversely, Au(110) displays much higher CO_2_R activities with a less positive PZC (Fig. 4, A to C). This discrepancy can be attributed to the unique characteristics of the Au(110) surface, which is known to expose highly active undercoordinated sites (CN = 7) compared to the relatively flat Au(100) and Au(111) terrace sites (CN = 9) (*36*). A positive correlation between the PZC value and CO_2_R activity is observed only between electrode surfaces with similar surface CNs, such as Au(100) and Au(111) terrace sites (CN = 9). This suggests that while there may be some relationship between PZC (or surface charge) and activity in specific cases, such correlations cannot be generalized across a broader range of catalysts. In conclusion, the observed structure-sensitive cation effects on CO_2_R activity cannot be solely explained by variations in PZC or surface charge density, highlighting the complex interplay of surface structure, coordination environment, and electrochemical activity in determining catalytic performance.

Factors beyond PZC or surface charge density, such as the interaction between cations and adsorbed CO_2_ intermediates, may play a more important role in influencing CO_2_R activity. Cations can selectively stabilize adsorbed CO_2_ or its formation transition state, leading to varied catalytic outcomes. For example, previous ab initio molecular dynamics simulations (*11*) suggest that Cs^+^ may more effectively stabilize adsorbed CO_2_ compared to Li^+^ on the same Au surface. This stronger electrostatic interactions between partially dehydrated Cs^+^-*CO_2_ complexes could enhance the overall CO_2_R activity. While the direct experimental measurement of these electrostatic interactions between hydrated cations and adsorbed CO_2_ is challenging, empirical observations can provide insights into such interactions before resorting to more detailed theoretical modeling with DFT. Therefore, experimental evidence is crucial in delineating these interactions, laying the groundwork for subsequent DFT calculations to comprehensively model and understand the mechanism driving cation-dependent CO_2_R reaction activity.

### Critical role of alkali metal cations in the electrokinetics of CO_2_R

**Fig. 5.**
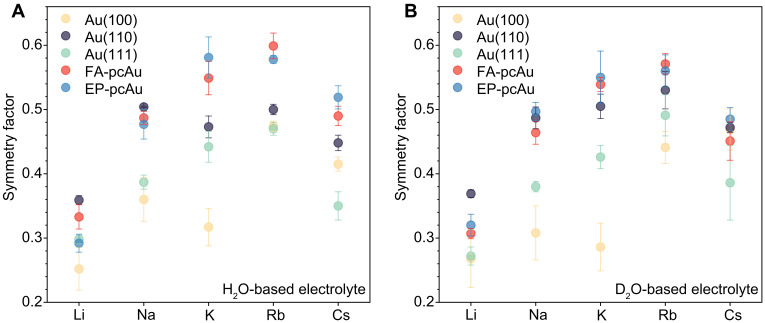
Values of symmetry factor (β) derived from Tafel plots of CO_2_R to CO as a function of cation identity. Values of β obtained in 0.1 M (**A**) H_2_O-based and (**B**) D_2_O-based bicarbonate electrolytes (pH = 6.8, *T* = 25 ± 1°C). Error bars (SDs) are calculated on the basis of three independent experiments.

Tafel analysis of CO_2_R under purely kinetic control on various Au electrodes in both H_2_O- and D_2_O-based electrolytes was performed, and the results are presented as symmetry factor (β) in Fig. 5 (see representative results in fig. S16). Note that only the transfer coefficient (α) can be derived from Tafel analysis for a multielectron transfer process, while β refers to a single-electron transfer step. However, measured α equals to β of the first step in this study because the first step is the RDS of CO_2_R on Au (see more details in Supplementary Note 1). A volcano-type relation was observed between the symmetry factor and the alkali cation across all Au electrodes in both H_2_O- and D_2_O-based electrolytes. This finding suggests that β values for CO_2_R are linked to the cations involved in the CO_2_ adsorption step (RDS). Specifically, the β value increases in the order of Li^+^ < Na^+^ < K^+^ < Rb^+^ and then decreases from Rb^+^ to Cs^+^. This trend holds consistently across the different Au electrodes, with Rb^+^ yielding the highest β values among all alkali metal cations on all Au surfaces except EP-pcAu. The similarity in β values observed between H_2_O- and D_2_O-based electrolytes further supports the notion that these effects are associated with the cation’s influence during the CO_2_ adsorption process where no proton transfer is involved.

The variation in β values with the surface structure adds another layer of complexity to understanding the cation effect. There is no overarching trend observed across different surface structures. For instance, Au(111) exhibits a higher β value compared to Au(100) in the K^+^ electrolyte, whereas the lowest β value is observed on Au(111) in the Cs^+^ electrolyte. This variability underscores the significance of considering electrode surface structures when assessing cation effects on CO_2_R. The distinct β values suggest that the interaction between cations and the CO_2_ adsorption process is influenced by the specific crystallographic orientation of the electrode surface. Therefore, the surface structure plays a crucial role in determining how cations modulate the kinetics and efficiency of CO_2_R on Au electrodes.

The CO_2_ adsorption process involving electron transfer can be represented by the equation

CO2+*+e−→*CO2− (1)

where * denotes an available surface site and *CO_2_^−^ represents an adsorbed CO_2_. Assuming that Butler-Volmer kinetics applies to this step in the absence of mass transport effects, the partial current density of CO ($j_{\text{CO}}$) can be described by

$$j_{\text{CO}}=2Fk^{\theta }a_{{\text{CO}}_{2}}\theta^{*}e^{\frac{-\beta \eta F}{\mathrm{RT}}}$$ (2)

where *k*^θ^ is the standard rate constant, $a_{{\text{CO}}_{2}}$ is the activity of CO_2_ near the electrode surface, θ* is the fraction of available surface sites, η is the overpotential, β is the symmetry factor (related to the electron transfer kinetics), *F* is the Faraday constant, *R* is the gas constant, and *T* is the temperature. In Eq. 2, it is assumed that the pre-exponential factor does not vary substantially with the cation on the same Au surface within the investigated potential window (variation less than one order of magnitude; see representative exchange current densities in table S10). When changing cations across the same Au surface structure at the same applied potential, the partial current density of CO is expected to increase as the β value increases. This correlation aligns with the observed volcano-type relation of β versus cation identity, where Rb^+^-containing electrolytes generally exhibit higher partial current densities of CO. The data also highlight the limitations of using a commonly simplified β value of 0.5 in kinetic modeling for CO_2_R, as it can lead to substantial uncertainties in estimating reaction activity and selectivity. Therefore, it is recommended to determine β values from experiments or additional theoretical modeling (*37*, *38*) to provide a more accurate assessment of cation effects in CO_2_R. These β values serve as sensitive indicators of how cations influence CO_2_R kinetics, particularly when PZC values remain relatively unchanged. The subsequent section delves into the molecular origins of β for the CO_2_ adsorption reaction and its dependence on electrical double-layer properties with DFT. This approach aims to provide deeper insights into the mechanisms underlying cation effects in CO_2_ electroreduction.

### Calculating the symmetry factor

DFT calculations were used to determine the electrokinetic symmetry factor β, focusing on the influence of cations and electrochemical double-layer (EDL) properties. Specifically, the process of CO_2_ adsorption and electron transfer to form *CO_2_^−^ was investigated, which is proposed to be the RDS of CO_2_R on Au electrodes (as discussed in Supplementary Note 1). In these calculations, EDL considerations were incorporated using a compartmentalized EDL theory model, as described in prior research (*39*). This model helps account for the activation barrier of the RDS in relation to the properties of the EDL, enhancing the understanding of how cations affect the kinetics of CO_2_ electroreduction.

The symmetry factor (β) is calculated by taking the first derivative of the activation barrier $\left(\Delta G_{\text{act}}\right)$ with reference to the applied potential (*U*)

$$\beta |e|\left(U\right)=\frac{\partial \Delta G_{\text{act}}\left(U\right)}{\partial U}=|e|+2\frac{\Delta \mu }{d}-\frac{\Delta \left(\alpha \mu \right)}{\varepsilon Ad^{2}}+\frac{\Delta \alpha }{d^{2}}\left(U-U_{\text{PZC}}\right)$$ (3)

Details of the derivations and calculation of the symmetry factor are discussed in Supplementary Note 2. The value of β is determined by properties of the EDL interface (ε and *d*) and the change in electronic properties of the surface-bound species (μ and α) between the initial and activated states. The dipole moment and polarizability changes (calculated along the surface normal direction) are calculated between the initial state [bare Au(111) surface or surface with (solvated) cation preadsorbed depending on the reaction path considered] and the transition state complex [including the adsorbing CO_2_ along with (solvated) cations depending on the reaction path]. Note that the |e| term appears in Eq. 3, where *a* + 1 cation (proton, alkali cation) is moved from the bulk electrolyte reference to the surface during the reaction being studied. In this case, the value of β is 1 if the interfacial dipole and polarizability changes [Δμ, Δ(αμ), and Δα] are 0 during the reaction process.

Equation 3 quantifies how the symmetry factor is sensitive to both the electronic character of the transition state and the approximation of the electrode-electrolyte interfacial properties (ε and *d* for the Helmholtz model used herein). Discussion of dominant variables when calculating β is discussed in Supplementary Note 2. Calculated values of β are strongly dependent on the magnitude of the dipole moment change and approximated EDL width. Extension of this approach for reaction models with explicit consideration of the alkali cations and H_2_O is denoted in Supplementary Note 2.1. A dielectric constant of 2 was used to represent the EDL as suggested by prior works (*40*, *41*).

### Reaction path models

The reaction path of electroreduction of CO_2_ to form a chemisorbed CO_2_^δ−^ species (*CO_2_^−^) is modeled on the Au(111), Au(100), and Au(110) surfaces. A series of local models of this reaction is considered, first neglecting any interaction of CO_2_ and the surface with water or alkali cations and then including explicit water molecules and alkali cations. As discussed below, a reaction pathway where (explicitly solvated) CO_2_ adsorbs without direct cation interaction is inconsistent with the experimentally observed β value, whereas electron transfer to a solvated CO_2_-cation complex is a plausible reaction model of CO_2_R because of consistency with the experimentally observed values of β.

### Formation of *CO_2_^−^ with explicit cations and H_2_O

**Fig. 6.**
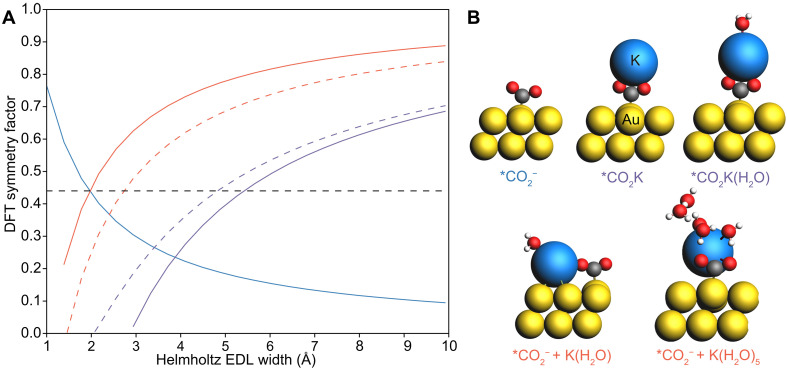
DFT modeling of formation of *CO_2_^−^ with explicit cations and H_2_O. (**A**) Profile of β_DFT_ with reference to EDL width from different transition state models of *CO_2_^−^ formation with explicit K* and H_2_O. The dash horizontal black line represents β_exp_ = 0.44 for the 0.1 M KHCO_3_ electrolyte on the Au(111) surface. The blue solid line is the profile of β_DFT_ for the simple *CO_2_^−^ model as seen in Fig. 7. The red solid and dashed lines represent the profiles of β_DFT_ for the coadsorbed cation models, respectively, for *CO_2_^−^ + *K(H_2_O) and *CO_2_^−^ + *K(H_2_O)_5_. The purple solid and dashed lines represent the profiles of β_DFT_ for the coordinated cation model, respectively, for *CO_2_-K and *CO_2_-K-H_2_O. A dielectric constant of 2 was chosen when calculating β_DFT_. (**B**) Optimized geometries of *CO_2_^−^ with explicit H_2_O and K* are shown. The structure of *CO_2_^−^ is derived from constrained geometries on the basis of COOH*. Atom colors are as follows: yellow, Au; blue, K; gray, C; red, O. Each activated complex label is color coded according to the lines in fig. S6A.

Supplementary Note 3 details that a simplistic model of the *CO_2_^−^ transition state lacks sufficient charge transfer from the metal to form a large enough magnitude of the dipole moment and therefore does not align with the experimentally observed values of β. Incorporating explicit cations and H_2_O into the DFT model for *CO_2_^−^ formation increases the dipole moment change along the reaction path by stabilizing additional charge transfer and/or placing that charge further from the metal surface. Reaction paths for the formation of *CO_2_^−^ with K^+^ and H_2_O explicitly considered were first studied on the Au(111) surface. Figure 6 illustrates different transition state models of *CO_2_^−^ interacting with an explicit alkali metal cation (K^+^) and explicit H_2_O. Calculated symmetry factors are compared with β_exp_ values obtained from experiments using 0.1 M KHCO_3_ on the Au(111) surface.

A sufficient dipole moment change upon the formation of the activated complex is needed to reconcile the experimentally observed symmetry factor values. The dipole moment change required for β_DFT_ = β_exp_ = 0.44, at an EDL width of 2.9 Å, is Δμ = −0.81 e^−^ Å (Eq. 3, with a cation involved) or Δμ = 0.67 e^−^ Å (eq. S11, without a cation involved). The simple model for *CO_2_^−^ (Δμ = −0.48 e^−^ Å) discussed in Supplementary Note 3 is shown by the blue line in Fig. 6A. *CO_2_^−^ without explicit cations or H_2_O molecules fails to provide sufficient dipole moment changes, thereby deviating from β_exp_.

In considering explicit K^+^ and H_2_O, decisions are made regarding the placement and number of ions and solvent molecules within the DFT model. In this work, we considered two cation-assisted transition state models: (i) a coadsorbed model [*CO_2_^−^ + *K(H_2_O)*_n_*] and (ii) a coordinated model [*CO_2_K(H_2_O)*_n_*]. Optimized geometries of these cation-assisted models are shown in Fig. 6B. The profile of β with reference to the EDL width for the coadsorption of a cation with its first hydration shell [K(H_2_O)_n_*] was considered and represented by the red solid and dashed line. Explicit H_2_O considerations are necessary when modeling *CO_2_^−^ with a coadsorbed K* since geometry optimization of the bent *CO_2_^−^ is unsuccessful without explicit H_2_O (fig. S24). Variations in the predicted dipole moment changes (resulting β_DFT_) are observed with the number and placement of explicit H_2_O molecules, as shown in table S6. However, regardless of the number of H_2_O molecules considered, the coadsorbed model is inconsistent with experimentally observed values of β. For β_DFT_ = β_exp_ = 0.44, unphysical EDL widths of 1.7 and 2.1 Å would be required for the respective transition state models of *CO_2_^−^ and K(H_2_O)_5_* and *CO_2_^−^ and K(H_2_O)*. This discrepancy arises as a consequence of the absolute magnitude of the dipole moment changes for the coadsorbed *CO_2_^−^ and K(H_2_O)_n_* models of the transition state, which is not sufficiently large enough, thus requiring unphysical values of the EDL width to agree with β_exp_ = 0.44.

Both the simple *CO_2_^−^ and coadsorbed cation models have an insufficient dipole moment change to agree with the experimentally observed symmetry factors, requiring the positive countercharge to be ~2 Å from the surface. This indicates that the cation must be close enough to interact and potentially coordinate with the surface-bound CO_2_^−^. Subsequent investigation explored the plausible transition states through a cation-coordinated model between *CO_2_^−^ and K^+^. The optimized geometries of the cation-coordinated models of the transition states are shown in Fig. 6B as *CO_2_K and *CO_2_K(H_2_O). β_DFT_ values of the cation-coordinated model, with and without an explicit H_2_O, are shown as the green dashed and solid lines in Fig. 6A. With the addition of one explicit H_2_O molecule, the profile of β_DFT_ with reference to EDL width is similar to the transition state model without any explicit H_2_O, where the differences arise from the polarizability change and not the dipole moment change.

Comparing with β_exp_, the β_DFT_ of the cation-coordinated models agrees with β_exp_ = 0.44 at an EDL width of 5.9 Å. The cation-coordinated model agrees with β_exp_ at physical EDL widths because of the larger dipole moment exhibited from the transition state (Δμ = −1.61 e^−^ Å for *KCO_2_). This suggests that the coordination between the explicit cation and *CO_2_^−^ within the DFT model is necessary for the DFT model to capture the symmetry factors observed experimentally. Tables S7 and S8 also show that the cation-coordinated model produces sufficiently large dipole moment changes across different cations (Li^+^, Na^+^, Rb^+^, and Cs^+^) on the Au(111) surface. These DFT results suggest that the transition state of *CO_2_^−^ is attained with explicit and direct interaction with alkali cations, where electron transfer occurs to this solvated CO_2_-cation complex during CO_2_R. This finding is consistent with observations by Koper and co-workers who noted that alkali cations were required in solution to facilitate CO_2_R (*11*) on Au through a coordinative mechanism.

The local DFT model used to examine the electron-accepting complex during CO_2_R does not necessarily capture the full range of states that may occur within a dynamic EDL. Sufficient sampling of a substantial number of ions and H_2_O molecules (>100) in a dynamic model of an electrochemical interface would require prohibitively long ab initio dynamic simulations (*39*, *42*, *43*). As a result, proposing a transition state that is stabilized by long-range interactions from multiple countercharge ions (electrostatic adsorption) or a larger network of H_2_O molecules is not feasible within a computationally tractable DFT periodic cell. Local static models, such as those used here, introduce uncertainty on the placement of ions and explicit H_2_O. Classical molecular dynamics can support the length and timescales of the electrode-electrolyte interface and provide reasonable sampling configurations of the electrolyte (*42*, *44*, *45*). However, molecular dynamics does not offer a straightforward approach for examining reactions involving electron transfer. Although these challenges prevent certainty that the models used here represent the exact reaction path, our calculated β_DFT_ values across local transition state models can offer insights into plausible mechanisms in comparison with experimental results. The cation-coordinated model emerges as the most reasonable model of the transition state during the formation of *CO_2_^−^. The sensitivity of the DFT model is shown through the various placements of the cation within the DFT model, which dictates the magnitude of the dipole moment changes and, ultimately, the symmetry factors.

### Trends across cations

**Fig. 7.**
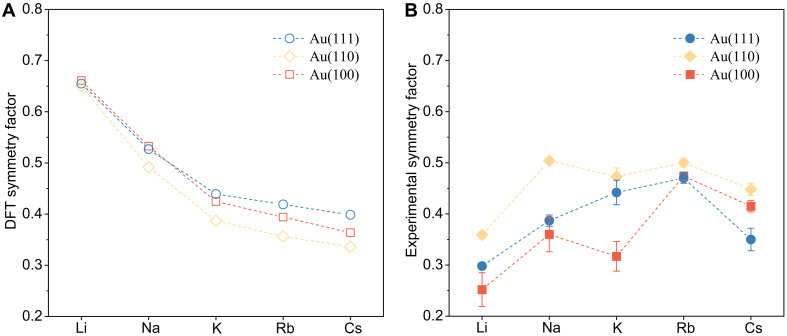
Comparison between DFT-predicted symmetry factor and experimental symmetry factor. (**A**) DFT-predicted symmetry factors across different alkali metal cations and surface facets of Au. The EDL width is assumed constant across alkali metal cations on the Au(111) surface. A Helmholtz EDL width of 5.9 Å was used, fitted to give β_DFT_ = β_exp_ for K^+^ on Au(111). A cation-coordinated model represents the transition state for *CO_2_^−^. (**B**) Experimentally derived symmetry factors across different alkali metal cations and surface facets of Au derived from Fig. 5.

Cation-coordinated transition states were systematically evaluated across different alkali cations and low-index Au facets [(111), (100), and (110)], with the goal correlating DFT-predicted symmetry factors with experimental trends. However, achieving consistent trends requires understanding how properties of the EDL (dielectric constant and effective countercharge distribution) vary with both the cation type and facet orientation. Figure 7 attempts such a comparison under the assumption that these EDL properties remain uniform, with values set calibrated to the DFT and experimental values observed for K/Au(111). In Fig. 7, it becomes evident that the trends across alkali cations and Au facets do not align between DFT predictions and experimental observations. This discrepancy may stem from challenges in accurately modeling the local interface or from assuming uniformity in the EDL properties across varying cations and facets. Further discussion of the potential origins of the disagreement between DFT and experiment is discussed in Supplementary Note 6. This disagreement between DFT and experiment underscores the unresolved mechanistic basis of the experimental trends. However, it strongly indicates that these trends arise from variations in EDL properties—specifically the effective dielectric response within the EDL region and the distribution of countercharges—that are dependent on both the alkali cation and Au facet under investigation. Elucidation of the double-layer properties is a fundamental challenge in electrocatalysis. Many DFT-based approaches have been developed to include features of the double layer, although they come with varying approximations and associated benefits or disadvantages in analyzing electrocatalysis phenomena. We refer the interested reader to the following book chapters or method development papers that provide review and perspective on these approaches, including considerations directly for CO_2_R (*39*, *46*–*48*).

## DISCUSSION

A comprehensive understanding of cation effects on CO_2_R is critical for developing a highly efficient electrode-electrolyte interface for building practical CO_2_R electrolyzers. To this end, CO_2_R was investigated on five model Au electrodes under well-defined mass-transport conditions in 0.1 M bicarbonate electrolytes (LiHCO_3_, NaHCO_3_, KHCO_3_, RbHCO_3_, and CsHCO_3_). Different cation trends were observed across the different Au electrodes, strongly suggesting that cation effects are structure sensitive and that the electrode surface structure plays an important role in cation effects on CO_2_R.

Although a more positive PZC favors higher CO_2_R activity on Au surface sites with comparable coordination numbers, PZC is a limited descriptor. Our Tafel analysis and KIE experiments suggest that the CO_2_ adsorption step, coupled with a concomitant electron transfer step, is most likely the RDS during CO_2_R on Au. Both the surface structure and cation identity can alter the symmetry factor of the RDS, with a volcano-type relation observed on almost all Au electrodes across different cations, where Rb^+^ gives the largest β values. DFT calculations further confirm that electron transfer occurs to a solvated CO_2_-cation complex in the RDS of CO_2_R. The symmetry factor of the RDS is sensitive to both the surface structure and EDL structure. Therefore, the observed structure-sensitive cation effects result from the interplay between the EDL structure and the electrode surface structure during CO_2_R. Both the electrode surface structure and EDL structure must be considered to explain cation effects, presenting a challenge for the electronic structure and atomistic modeling of CO_2_R.

## MATERIALS AND METHODS

### Chemicals

The electrolyte solutions were prepared from H_2_SO_4_ (99.999% purity, Sigma-Aldrich), Li_2_CO_3_ (99.999%, Thermo Scientific Chemicals), Na_2_CO_3_ (99.999%, Thermo Scientific Chemicals), K_2_CO_3_ (99.997%, Thermo Scientific Chemicals), Rb_2_CO_3_ (99.975%, Thermo Scientific Chemicals), Cs_2_CO_3_ (99.994%, Thermo Scientific Chemicals), K_3_Fe(CN)_6_ (99%, Sigma-Aldrich), D_2_O (99.9 atom % D, Sigma-Aldrich), Chelex-100 (sodium form, 50 to 100 mesh, Sigma-Aldrich), and ultrapure water (Millipore Milli-Q, 18.2 megohm·cm, total organic carbon of <3 parts per billion). Ar (99.998%, Linde), CO (99.99%, Linde), CO_2_ (99.999%, Linde), and H_2_ (99.995%, Linde) were used for purging the electrolyte solutions.

### Single-crystal electrode preparation

Gold single-crystal disk electrodes (99.999%; diameter, 5 mm; thickness, 4 mm) were purchased from Princeton Scientific. All single-crystal electrodes were cut with a nominal orientation accuracy smaller than 0.1° and the polishing roughness smaller than 10 nm. Before each independent measurement, the electrodes were flame annealed to red heat for ~10 s with a propane torch (note that the room light should be turned off for a better observation of the temperature color) and cooled down to room temperature in an argon stream (~30 min). Then, the polished single-crystal surface was attached to a droplet of Milli-Q water and carefully transferred to a clean polytetrafluoroethylene mounting block to assemble the RRDE tip.

### EP-pcAu disk and ring electrode preparation

Before each experiment, the gold disk (99.99%; diameter, 5 mm; thickness, 4 mm; Pine Instruments) and ring electrodes (geometric surface area ≈ 0.110 cm^2^; 99.99%, Pine Instruments) were mechanically polished on Buehler polishing cloth with decreasing sizes of 1-, 0.25-, and 0.1-μm diamond suspensions (Electron Microscopy Sciences). Next, the RRDE tip was sonicated three times in acetone and ultrapure water (3 min each) to remove any impurities attached to the RRDE tip. Then, the RRDE tip was transferred to the electrochemical polishing cell, and the gold disk electrode and gold ring electrode were short circuited and cycled between 0.05 and 1.75 V_RHE_ for 150 cycles at 1 V/s in 0.1 M argon-saturated H_2_SO_4_. A representative CV of the Au ring electrode is shown in fig. S2.

### FA-pcAu disk electrode preparation

Before each experiment, the gold disk (99.99%; diameter, 5 mm; thickness, 4 mm; Pine Instruments) was mechanically polished on Buehler polishing cloth with decreasing sizes of 1-, 0.25-, and 0.1-μm diamond suspensions (Electron Microscopy Sciences). Next, the RRDE tip was sonicated three times in acetone and ultrapure water (3 min each) to remove any impurities attached to the RRDE tip. Then, the gold disk was disassembled from the RRDE tip, flame annealed to red heat for ~10 s with a propane torch, and cooled down to room temperature in an argon stream. Then, the gold disk surface was attached with a droplet of Milli-Q water and carefully transferred to a polytetrafluoroethylene mounting block to assemble the RRDE tip.

### Purification of the H_2_O-based electrolyte

To prepare prepurified electrolyte solutions with ultralow content of polyvalent metallic impurities, M_2_CO_3_(M = Na, K, Rb, and Cs; trace metal basis) and disposable polystyrene spoons (VWR) were used to prepare a 0.05 M M_2_CO_3_ solution with ultrapure water in a clean polystyrene plastic bottle to avoid any possible trace metal contamination from the glassware, then ~30 g of Chelex-100 per mole of M_2_CO_3_ was added to the solution, and then this mixture was stirred at 700 rpm for 24 hours. Any glassware should be avoided during the long-term (≥24 hours) prepurification process because of the possible leaking of Si^4+^ from glass corrosion in alkaline M_2_CO_3_ solution. Next, insoluble Chelex-100 resin particles were removed from the electrolyte solution by vacuum filtration with the help of a polyethersulfone membrane (pore size, 0.45 μm; VWR). The filtered solution volume was adjusted using a 1-liter polymethylpentene plastic volumetric flask (BRAND), and the ultrapure electrolyte solution was stored in a clean polystyrene bottle.

In the case of 0.05 M Li_2_CO_3_, the pre-electrolysis method was adopted instead of Chelex-100 because Chelex-introduced Na^+^ may support non-negligible CO_2_R activity. Briefly, the 0.05 M Li_2_CO_3_ electrolyte was pre-electrolyzed at a constant reducing current of −10 mA for ~24 hours with a stirring rate of 700 rpm in a Nafion membrane–separated (Nafion 117, Sigma-Aldrich) two-compartment cell. The gold foil (2.5 cm by 2.5 cm, 99.99%, Thermo Scientific Chemicals) and a graphite rod (99.9995%, Thermo Scientific Chemicals) were used as working and counter electrodes, respectively. Gold wires (99.999%, Sigma-Aldrich) were used to build the electrical contact between the electrodes and the potentiostat. Before finishing the pre-electrolysis process, the working electrode was taken out from the solution under the potentiostat control to avoid possible oxidation of deposited metal impurities at open-circuit voltage.

Before each independent experiment, 100 ml of 0.05 M carbonate solution was bubbled through CO_2_ for 1 to 2 hours with a flow rate of 120 SCCM (standard cubic centimeters per minute) using a mass flow controller (Sierra Instruments), and the pH of the solution was verified to be around 6.8 by using a calibrated pH meter (XL20, Thermo Fisher Scientific), which indicates the complete conversion from a 0.05 M carbonate solution to a 0.1 M CO_2_-saturated bicarbonate solution. The electrolyte was checked using electrochemical characterizations described in a previous work (*49*).

### Purification of the D_2_O-based electrolyte

It has been reported that even commercial D_2_O with the highest purity cannot be used as received for accurate KIE measurement (*50*). To purify the D_2_O-based electrolyte, the commercial D_2_O solvent was illuminated for 6 hours under ultraviolet light using a dual-wavelength ultraviolet lamp (185 nm/254 nm, 25 W) to remove organic carbon contamination. Then, the same procedures as purifying the H_2_O-based electrolyte were followed to remove potential polyvalent metallic impurities from the D_2_O-based electrolyte.

### General electrochemical measurements

All the electrochemical measurements were carried out in homemade standard three-electrode glass cells (volume ≈ 180 ml). The counter electrode was a high-purity graphite rod (99.9995%, Thermo Scientific Chemicals) separated from the working compartment with a porous glass frit. Either a homemade reversible hydrogen electrode (RHE) or a leak-free Ag/AgCl reference electrode (3.4 M KCl, Innovative Instruments) was used as the reference electrode, which was separated from the working compartment through a homemade Luggin capillary. All the glassware was rinsed and sonicated before each independent experiment with diluted Piranha solution and boiling ultrapure water three times (3 min each), and we note that sonication is necessary because contaminants accumulated in glass frits are hard to be removed completely by rinsing only. For all measurements, 85% ohmic drop compensation was performed using a CHI 760D bipotentiostat (CH Instruments), and the remaining 15% drop was compensated manually after the experiment. The ohmic drop of the system was determined by carrying out potentiostatic electrochemical impedance spectroscopy (PEIS) at 0.10 V_RHE_ (Δ*V* = 10 mV, from 100 kHz to 1 Hz). The ohmic resistance of the cell (*R*_u_) was obtained by extrapolation of the fitted modified Randles equivalent circuit with a constant phase element as shown in fig. S10. The measured potentials were converted to the RHE scale by *E*_RHE_ = *E*_Ag/AgCl_ + 0.210 V + 0.059 V × pH.

### PZC determination

To determine the values of PZC, additional PEIS measurements (Δ*V* = 10 mV, from 100 kHz to 1 Hz) were carried out in CO_2_-saturated 1 mM MHCO_3_ (pH = 4.8; M = Li, Na, K, Rb, and Cs) electrolytes with a rotation rate of 1600 rpm using a Gamry reference 600+ potentiostat. The double-layer capacitance was determined by fitting the PEIS data with a modified Randles equivalent circuit as shown in fig. S10. Briefly, *C*_dl_ was directly derived from a constant phase element term [*Z*_CPE_ = *C*_dl_′^−1^(*j*ω)^−*n*^], and we assume that *C*_dl_′ represents the true double-layer capacitance when the *n* value is larger than 0.90, which indicates that the double layer presents pure capacitive behavior without showing notable frequency dispersion behavior. As shown in fig. S11, the potential of global double-layer capacitance minimum in the 1 mM bicarbonate electrolyte corresponds to the PZC. Tabulated PZC values are shown in table S1.

### RRDE experiments

To perform CO_2_R study using an RRDE setup, the disk and ring electrodes were prepared as mentioned before. The measurements were conducted in a 0.1 M CO_2_-saturated MHCO_3_ electrolyte solution (pH = 6.8, *T* = 25 ± 1°C). The disk electrodes were immersed in the electrolyte under potential control at 0.10 V_RHE_, while the ring electrode was cycled between 0.05 and 1.75 V_RHE_ for five cycles at 50 mV s^−1^. Then, the ring electrode potential was set to 0.98 V_RHE_ and the disk electrode was scanned between 0.10 and 0 V_RHE_ at 15 mV s^−1^ (or 5 mV s^−1^ for obtaining Tafel plots) for 10 cycles to allow the ring current background to reach a steady-state value; the disk was scanned to more negative potentials at 15 mV s^−1^ to conduct CO_2_R, while the ring potential was set to 0.98 V_RHE_ once a stable ring background current was recorded. To derive Tafel slopes from CO_2_R polarization curves measured from the ring currents, a lower scan rate of 5 mV s^−1^ was used to achieve a better signal-to-noise ratio and the potential window between −0.35 and −0.44 V_RHE_ was chosen as the fitting region of the Tafel plot for the RDS. The collection efficiency was measured immediately after the RRDE experiment and voltammetric surface characterization. The reported results were averaged on the basis of three independent experimental measurements, in which freshly prepared electrolytes and electrodes were used. At potentials more negative than −0.50 V_RHE_ with a scan rate of 15 mV s^−1^, the RRDE tip suffered from a bubble attachment issue because of a higher total current density, which was especially evident in Rb^+^- and Cs^+^-containing electrolytes; thus, the most negative potential was chosen to be −0.50 V_RHE_.

To further ensure the validity of the RRDE voltammetry method in studying the electrokinetics of CO_2_R, scan-rate dependent voltammetry and chronoamperometry of CO_2_R experiments were conducted as shown in figs. S5 and S6, respectively. There is no detectable major difference in current densities upon changing scan rates (15 to 1 mV s^−1^) or changing from cyclic voltammetry to chronoamperometry; thus, it is safe to assume that our measured currents approach the steady state.

### Collection efficiency determination

After each RRDE experiment, the apparent collection efficiency (N) was determined in a separate cell containing 0.1 M Ar-saturated NaHCO_3_ with 10 mM K_3_Fe(CN)_6_. The disk was cycled between 0.30 and 1.20 V_RHE_, and the ring potential was set to 1.20 V_RHE_; representative results are shown in fig. S4. The collection efficiency was calculated according to Eq. 4

$$N=\left|\frac{i_{\text{ring}}}{i_{\text{disk}}}\right|$$ (4)

### Electrochemical surface area determination

After CO_2_R experiment, characterization cyclic voltammetry of the disk electrode was obtained between 0.05 and 1.75 V_RHE_ at a scan rate of 50 mV s^−1^. The electrochemically active surface area of the pcAu electrode was determined by calculating the total charge from integrating the reduction peak in the characterization CV and dividing it by the specific charge, which corresponds to the reduction of one monolayer of gold monoxide (386 μC cm^−2^) (*51*).

### RRDE data processing

The partial current density of CO evolution is calculated from the background-subtracted ring current ($i_{\text{ring}}$), the apparent collection efficiency (N), and the electrochemically active surface area of the disk (${\text{ECSA}}_{\text{disk}}$)

$$j_{\text{CO}}=\frac{-i_{\text{ring}}}{N\times {\text{ECSA}}_{\text{disk}}}$$ (5)

Then, the faradic efficiency for CO formation can be calculated as

$${\text{FE}}_{\text{CO}}=\left|\frac{i_{\text{ring}}}{i_{\text{disk}}\times N}\right|\times 100\%$$ (6)

where $i_{\text{disk}}$ is the experimentally obtained total current on the disk.

### Symmetry factor (β) value determination

The Tafel slopes were determined from a straight-line region in the Tafel plots, and the fitted Tafel slopes are considered to be valid only when they are independent of enhanced mass transport rate (variation, <5%), which indicates that the CO_2_R activity is under purely kinetic control. Typical Tafel plots are shown in figs. S16 and S17. The valid Tafel slopes were converted to symmetry factor values as below

$$\beta =\frac{59.2\;\text{mV}\;\text{per}\;\text{decade}}{\text{Tafel slope}}$$ (7)

and the measurements were repeated three times independently to ensure that the derived values are statistically meaningful.

### Electronic structure calculations

The Vienna Ab Initio Simulation Package was used for all electronic structure calculations in this work (*52*–*55*). The projector augmented wave method was used to approximate the core-valence electrons (*56*, *57*). The Perdew, Burke, and Ernzerhof exchange correlation functional within the generalized gradient approximation was used for all calculations (*58*–*60*). An energy cutoff value of 450 eV was used to truncate the plane wave basis set. The ionic convergence limit was set to 0.05 eV Å^−1^ (EDIFFG), while the electronic convergence limit was set to 10^−5^ eV (EDIFF). A Fermi smearing width of 0.2 (SIGMA) was used for surface-bound species on the basis of the Methfessel-Paxton scheme (*61*). Additional *s* semi-core valence electron states are treated for all alkali metal cations.

### Model construction

The Au(111), Au(110), and Au(100) surface facets were modeled as five-layer 3 by 3 periodic surface slabs. The top two layers were relaxed to undergo surface reconstruction, while the bottom three layers were constrained to represent the bulk material. A 15-Å vacuum region was applied across all surface facets. Dipole moments within the surface model were calculated by applying a dipole correction in the normal direction of the periodic surface (IDIPOL = 3; LDIPOL = .TRUE.). A Monkhorst-Pack method was used to sample the Brillouin zone (*62*, *63*). A 5 by 5 by 1 *k*-point mesh grid was used for the Au(100) surface, a 7 by 7 by 1 *k*-point mesh grid for the Au(110) surface, and a 11 by 11 by 1 *k*-point mesh grid for the Au(111) surface.

## Supplementary Materials

### This PDF file includes:

Supplementary Notes 1 to 6 Figs. S1 to S36 Tables S1 to S11 References
