# Supplementary material for: Cation effects on CO2 reduction catalyzed by single-crystal and polycrystalline gold under well-defined mass transport conditions
Source: Sci Adv. 2025 Feb 7;11(6):eadr6465. doi: 10.1126/sciadv.adr6465 (PMC11804923; doi:10.1126/sciadv.adr6465)
Supplement: Supplementary file 1 — Supplementary Notes 1 to 6 Figs. S1 to S36 Tables S1 to S11 References [file sciadv.adr6465_sm.pdf]

Science Advances
AAAS

Supplementary Materials for

Cation effects on CO_2 reduction catalyzed by single-crystal and polycrystalline gold under well-defined mass transport conditions

Zhihao Cui *et al.*

Corresponding author: Anne C. Co, co.5@osu.edu; Michael J. Janik, mjanik@psu.edu

Sci. Adv. **11**, eadr6465 (2025)
DOI: 10.1126/sciadv.adr6465

This PDF file includes:

Supplementary Notes 1 to 6
Figs. S1 to S36
Tables S1 to S11
References

Supplementary Note 1. Evidence for the RDS of CO_2R from Kinetic Isotope Effect (KIE) experiments and Tafel analysis

To further confirm that the rate determing step (RDS) of CO_2R is CO_2 adsorption with a concomitant electron transfer, for all surface structures and cation identities, the CO partial current densities were measured using RRDE voltammetry method in D_2O-based 0.1 M $MDCO_3$ (M = Li, Na, K, Rb and Cs) electrolytes. The results are shown in **Fig. S12 (f-j)** for comparison with the results obtained in H_2O-based electrolytes shown in **Fig. S12 (a-e)**. Through replacing hydrogen with deuterium, it was previously reported that a KIE of ca. 7 was observed during homogeneous CO_2 reduction to CO on an iron porphyrin catalyst (*64*). We would expect a KIE > 1 if the RDS invovles a proton transfer during CO_2R. Our results show that partial current densities of CO are very similar (KIE ≈ 1) in both H_2O-based and D_2O-based electrolytes, which would suggest that the RDS does not involve a proton transfer on all Au electrodes in different electrolytes. Combining this important evidence with our fitted Tafel slopes (from ca. 100 to 280 mV/dec, which are consistent with previous published results (*65, 66*), representative results are shown in **Fig. S16 and S17**) from a fitting range between -0.35 and -0.44 V_{RHE} obatained on various Au electrodes, we conclude that the CO_2 adsorption with a concomitant electron transfer step is most likely to be the RDS during CO_2 reduction when the electrode potential is more negtaive than -0.35 V_{RHE}, regardless of cation identity and the surface structure. This conclusion agrees with previous work from different groups (*11, 34, 35, 67, 68*) which also determined the adsorption of CO_2 with a concomitant electron transfer step to be the RDS during CO_2R. These results also suggest that cation-dependent reorganization of interfacial water is unlikely to account for observed cation effects in this work, as the

RDS does not involve a proton transfer which is expected to be insensitive to interfacial water structure change.

However, it is important to point out that complications exist in probing KIE effects in D_2O-based electrolytes for CO_2R (*34, 69*). A measured KIE ≈ 1 could also indicate a RDS to be the $^*COOH$ to $^*CO$ step through a concerted proton coupled electron transfer, as the change of zero point energy of this step is negligible through replacing H_2O with D_2O. We can exclude this possibility in this work as Tafel slopes should be smaller than 59.2 mV/dec if the second proton-electron transfer step limits the overall reaction. Thus, KIE results should be combined with other experimental evidence such as Tafel analysis or pH-dependence study to draw more reliable conclusions about the RDS of CO_2R.

Although the partial current densities of CO are similar, it is interesting to note that the Faradaic efficiencies of CO obtained in D_2O-based electrolytes (**Fig. S15**) were higher than those obatained in H_2O-based electrolytes (**Fig. S9**), which is attributed to a lower activity of the competitive hydrogen evolution reaction (HER) in D_2O-based electrolytes (*69*).

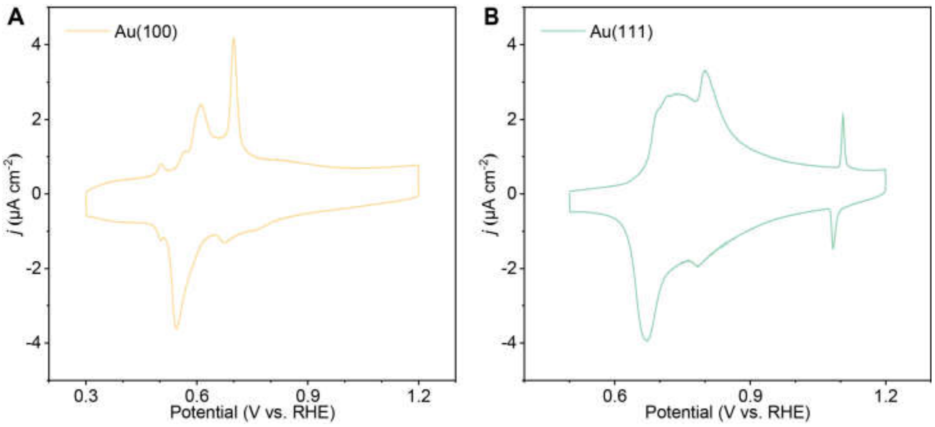

Fig. S1. Cyclic voltammetry characterization of Au(100) and Au(110) electrodes. Cyclic voltammograms of **(A)** Au(100) and **(B)** Au(111) recorded in the double layer region in Ar-saturated 0.1 M H_2SO_4 with a scan rate of 20 mV s^{-1}.

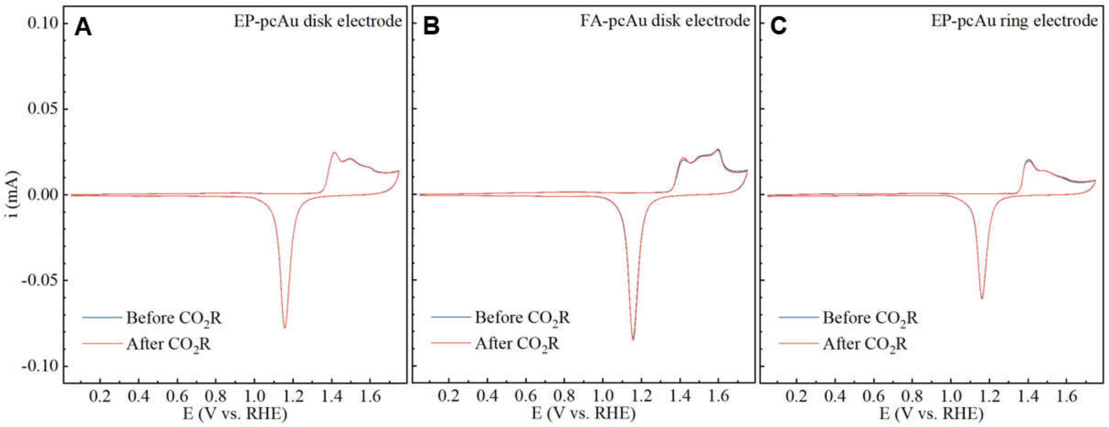

Fig. S2. Cyclic voltammetry characterization of polycrystalline Au electrodes before and after CO_2R. Cyclic voltammograms of **(A)** electrochemically polished Au disk electrode, **(B)** flame-annealed Au disk electrode and **(C)** electrochemically polished Au ring electrode recorded in Ar-saturated 0.1 M H_2SO_4 with a scan rate of 50 mV s^{-1}.

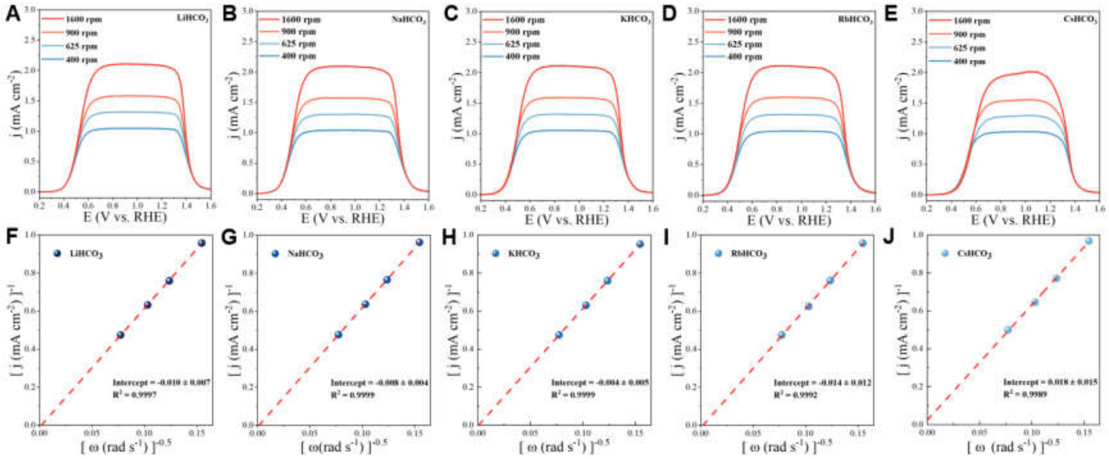

Fig. S3. Koutecky-Levich analysis for CO oxidation on Au. (A-E) CO oxidation CVs on Au ring at different rotation rates in CO-saturated 0.1 M bicarbonate electrolyte (pH = 8.9), recorded at 15 mV s^{-1} from 0.2 to 1.6 V_{RHE}, only positive scans were shown for clarity (The variation of the limiting current density is smaller than 5% in different electrolytes). **(F-J)** Koutecky-Levich analysis for CO oxidation current densities at 0.98 V_{RHE} in corresponding electrolytes (Note that Koutecky-Levich analysis was originally developed for the rotating disk electrodes, our goal is to show CO oxidation reached mass transport limited currents on a freshly prepared Au ring electrode in different 0.1 M bicarbonate electrolytes, not to derive the exact values of kinetic parameters).

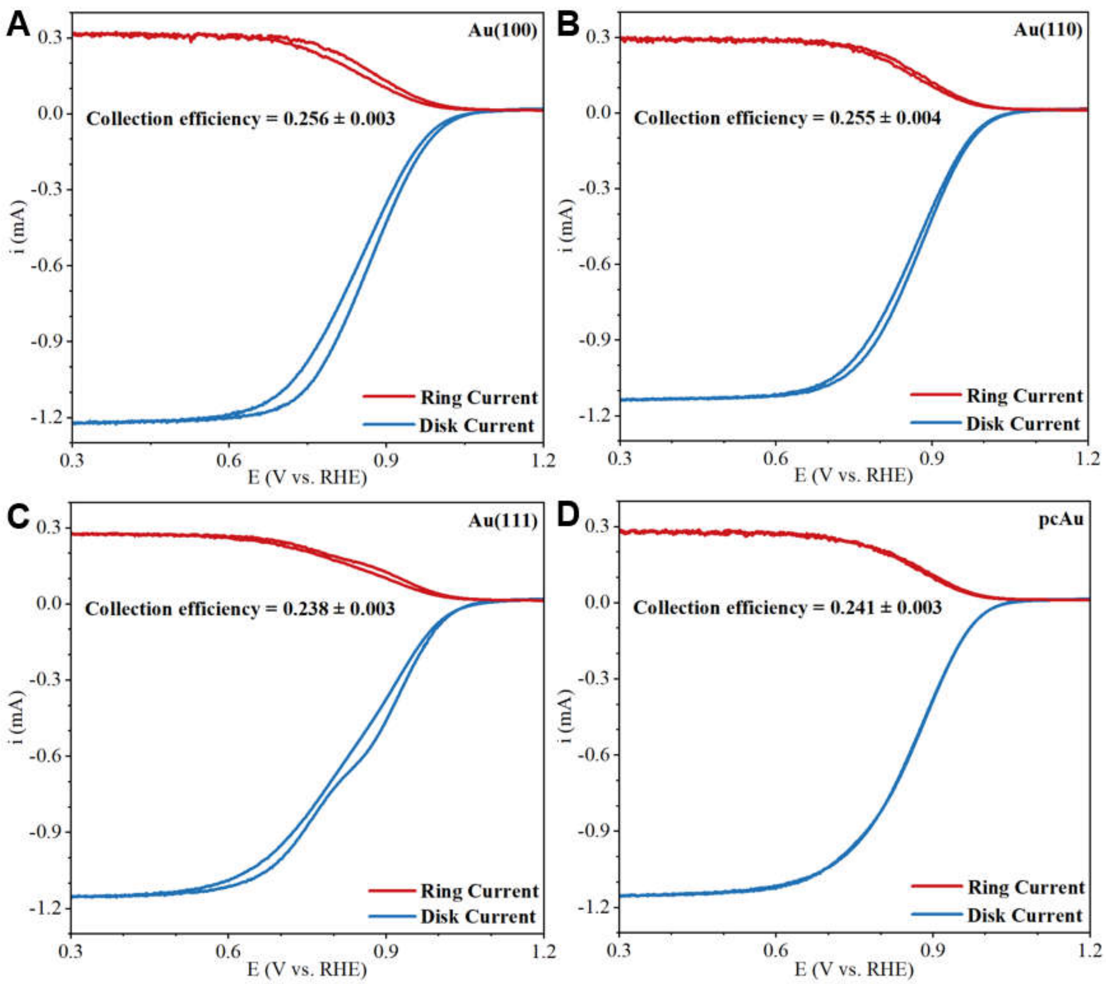

Fig. S4. Collection efficiency determination experiments. CVs recorded on **(A)** Au(100), **(B)** Au(110), **(C)** Au(111) and **(D)** pcAu in 10 mM $K_3Fe(CN)_6$ contained in 0.1 M $NaHCO_3$ with a scan rate of 15 mV s^{-1} at 1600 rpm.

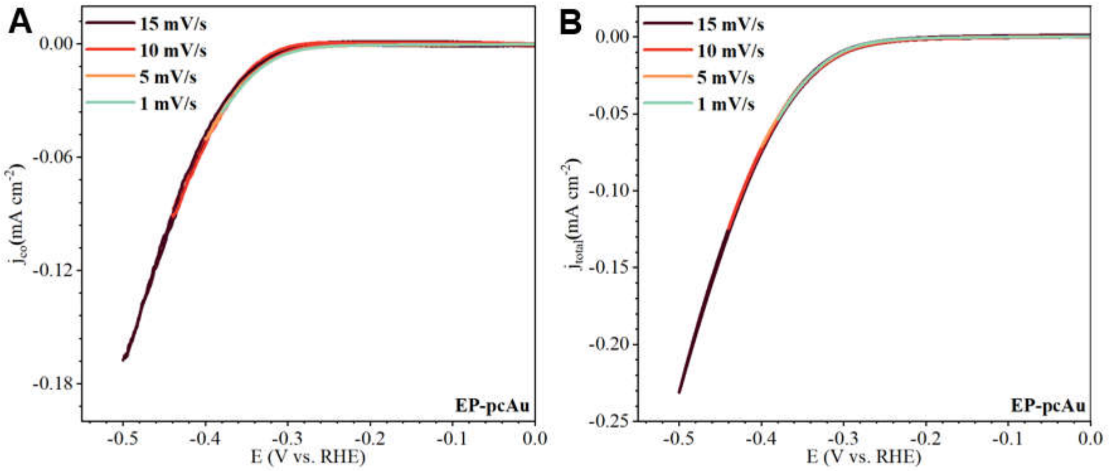

Fig. S5. Scan rate dependence experiments. (A) The partial current densities of CO and **(B)** total current densities measured by RRDE voltammetry at 1600 rpm with different scan rates in CO_2-saturated 0.1 M $NaHCO_3$ (pH=6.8).

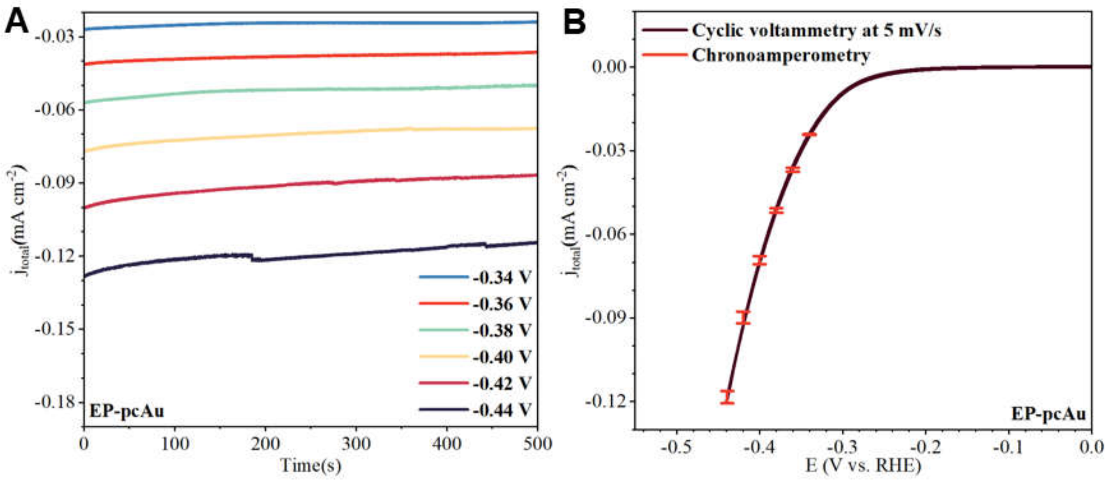

Fig. S6. A comparison between chronoamperometry and cyclic voltammetry. (A) Total current densities obatined at indicated potentials in CO_2-saturated 0.1 M $NaHCO_3$ by RRDE chronoamperometry (CA) at 1600 rpm. **(B)** A comparison between averaged CA current densities with CV current densities recorded in CO_2-saturated 0.1 M $NaHCO_3$ (pH=6.8) at a scan rate of 5 mV s^{-1}.

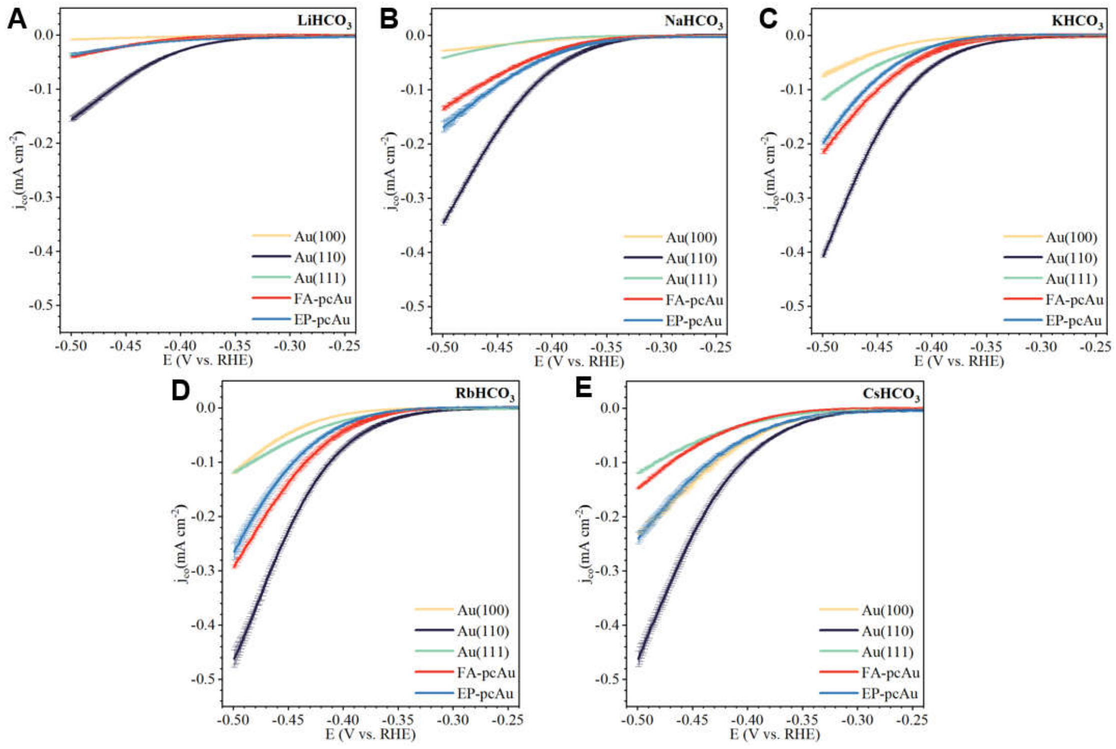

Fig. S7. Determination of the CO partial current densities. The CO partial current densities measured by RRDE voltammetry in 0.1 M **(A)** $LiHCO_3$, **(B)** $NaHCO_3$, **(C)** $KHCO_3$, **(D)** $RbHCO_3$ and **(E)** $CsHCO_3$ during CO_2R at 15 mV s^{-1} and 1600 rpm in CO_2 saturated 0.1 M bicarbonate electrolytes (pH=6.8).

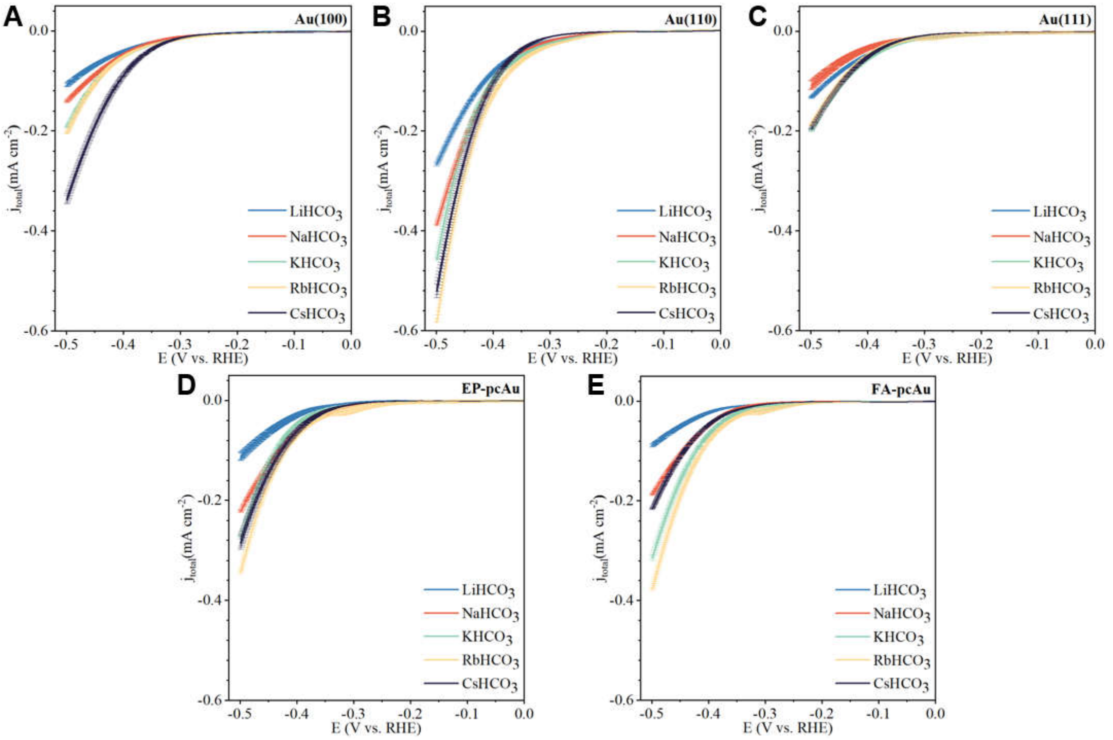

Fig. S8. Determination of the total current densities. The total current densities measured by RRDE voltammetry on **(A)** Au(100), **(B)** Au(110), **(C)** Au(111), **(D)** EP-pcAu and **(E)** FA-pcAu during CO_2R at 15 mV s^{-1} and 1600 rpm in CO_2 saturated 0.1 M bicarbonate electrolytes (pH=6.8).

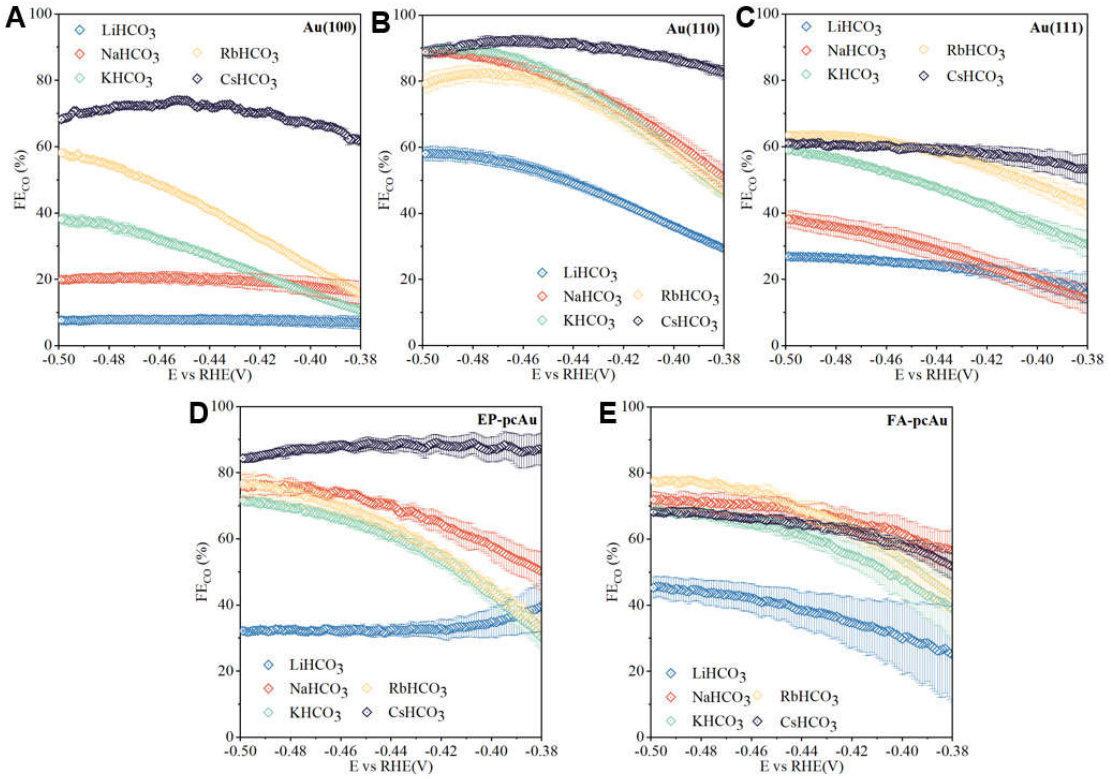

Fig. S9. Determination of the Faradic efficiencies. The Faradic efficiencies of CO calculated from RRDE voltammetry data on **(A)** Au(100), **(B)** Au(110), **(C)** Au(111), **(D)** EP-pcAu and **(E)** FA-pcAu during CO_2R at 15 mV s^{-1} and 1600 rpm in CO_2 saturated 0.1 M bicarbonate electrolytes (pH=6.8).

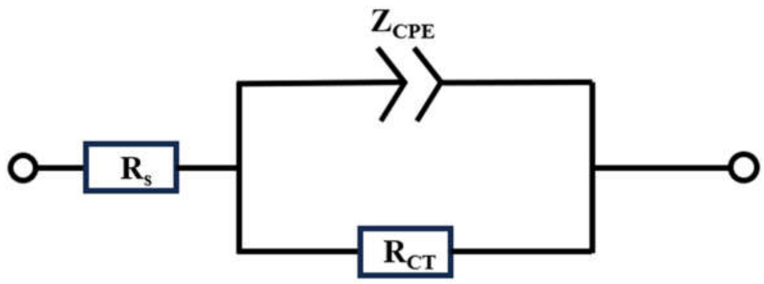

Fig. S10. A schematic of the equivalent circuit. Modified Randles equivalent circuit with a constant phase element (CPE). Z_{CPE}, R_s and R_{CT} are constant phase element term, solution resistance and charge transfer resistance, respectively (Note that the R_{ct} term is negligible and the derived capacitance comes from the Z_{CPE} term in the double-layer potential window).

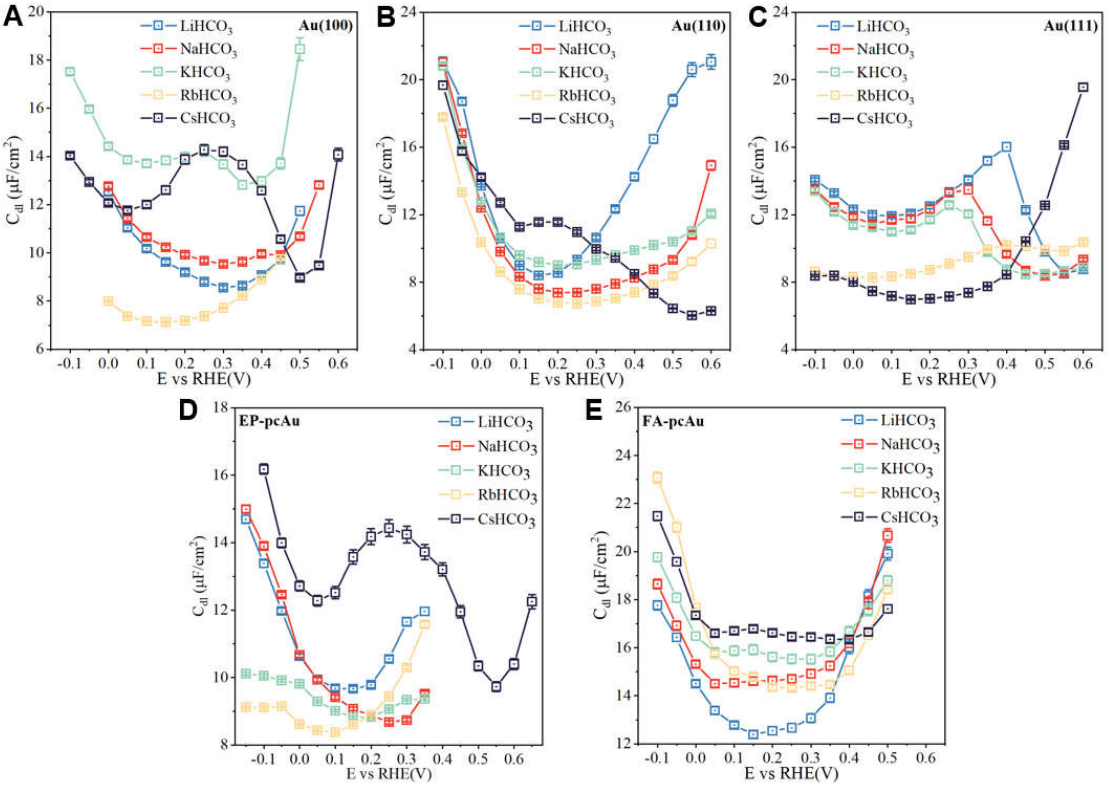

Fig. S11. PZC determination experiments. Measured double layer capacitance (C_{dl}) by potentiostatic electrochemical impedance spectroscopy (PEIS) on **(A)** Au(100), **(B)** Au(110), **(C)** Au(111), **(D)** EP-pcAu and **(E)** FA-pcAu at double layer region with a rotation rate of 1600 rpm in CO_2 saturated 1 mM bicarbonate electrolytes (pH=4.8).

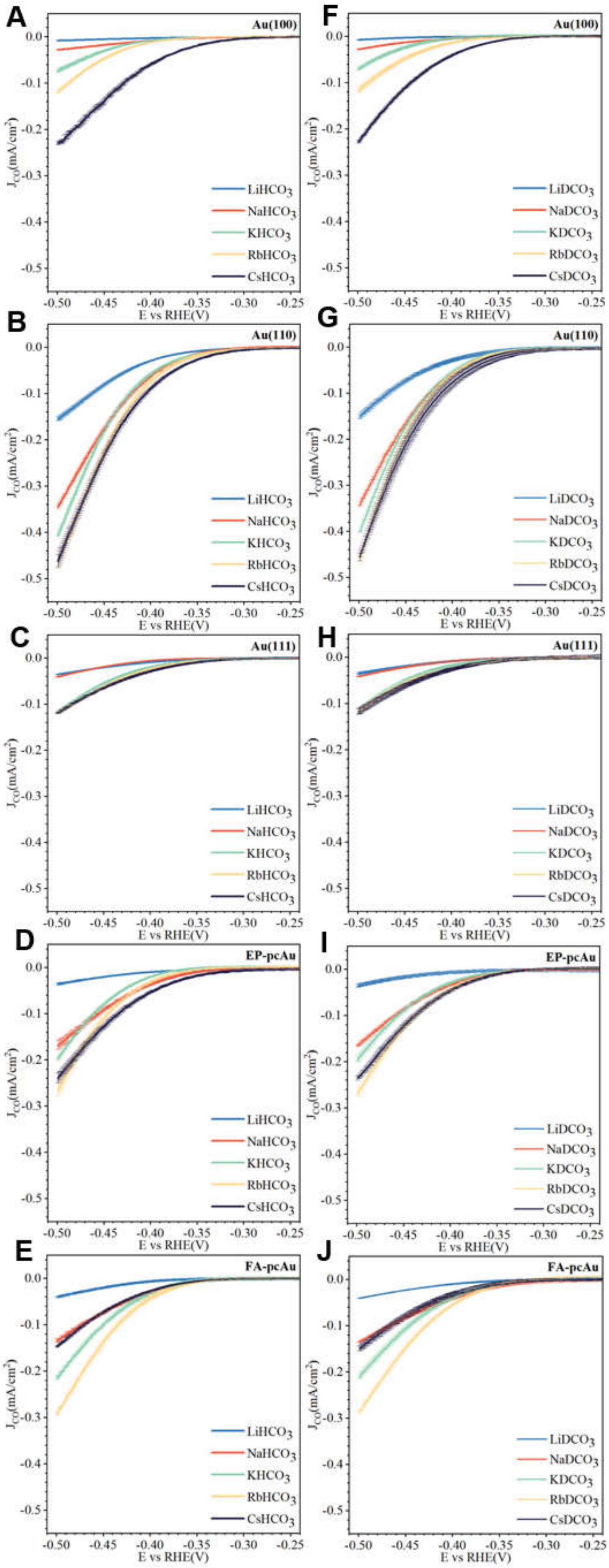

Fig. S12. Kinetic isotope effect experiments. The CO partial current densities measured by RRDE voltammetry in **(A-E)** H_2O-based electrolytes and **(F-J)** D_2O-based electrolytes during CO_2R at a scan rate of 15 mV s^{-1}.

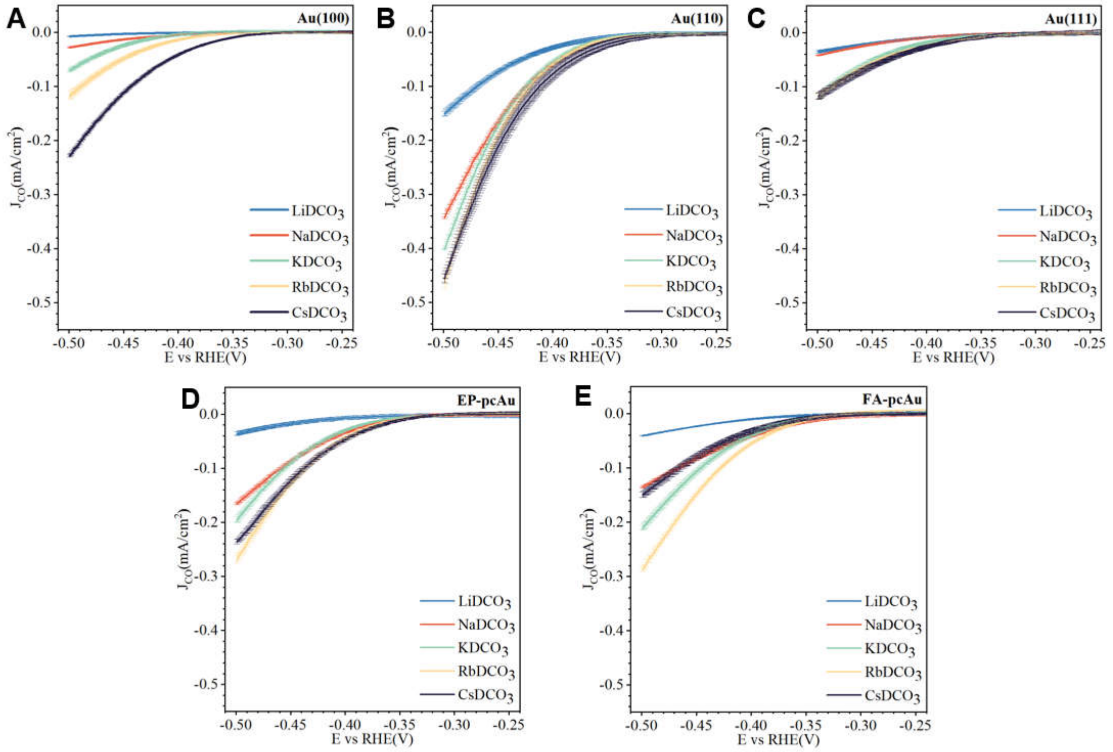

Fig. S13. Determination of the CO partial current densities. The CO partial current densities measured by RRDE voltammetry on (**A**) Au(100), (**B**) Au(110), (**C**) Au(111), (**D**) EP-pcAu and (**E**) FA-pcAu during CO_2R at 15 mV s^{-1} and 1600 rpm in CO_2 saturated D_2O bsed 0.1 M bicarbonate electrolytes (pH=6.8).

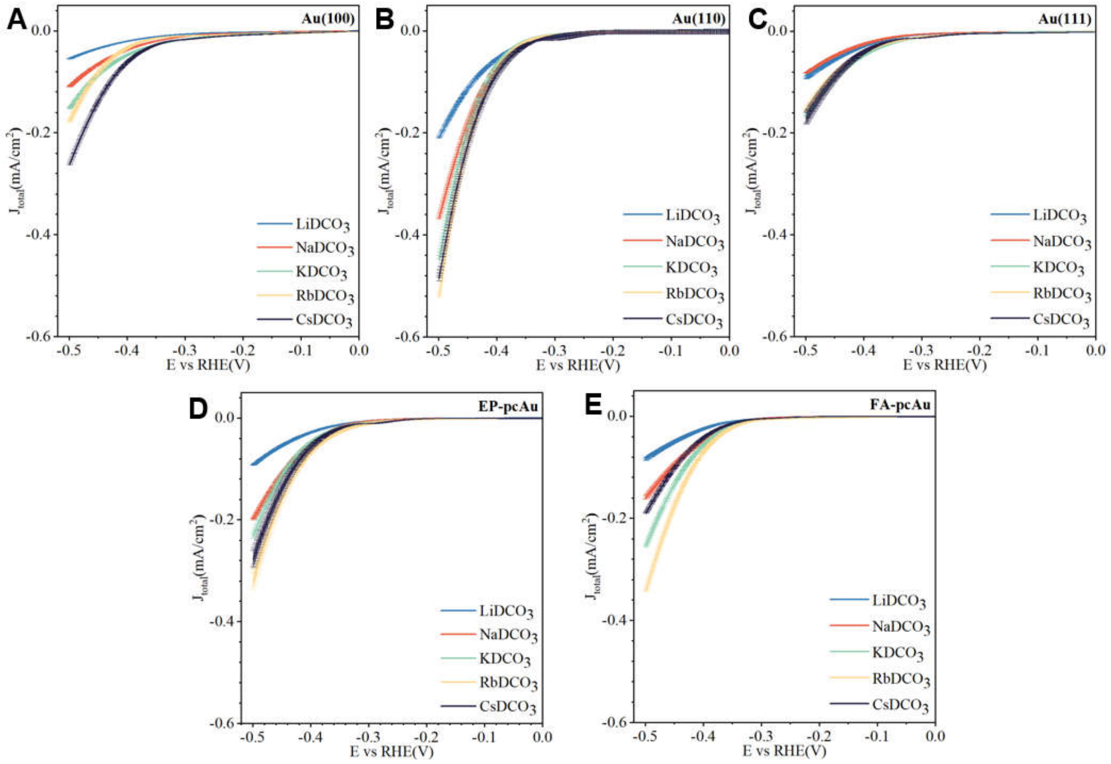

Fig. S14. Determination of the total current densities. The total current densities measured by RRDE voltammetry on **(A)** Au(100), **(B)** Au(110), **(C)** Au(111), **(D)** EP-pcAu and **(E)** FA-pcAu during CO_2R at 15 mV s^{-1} and 1600 rpm in CO_2 saturated D_2O based 0.1 M bicarbonate electrolytes (pH=6.8).

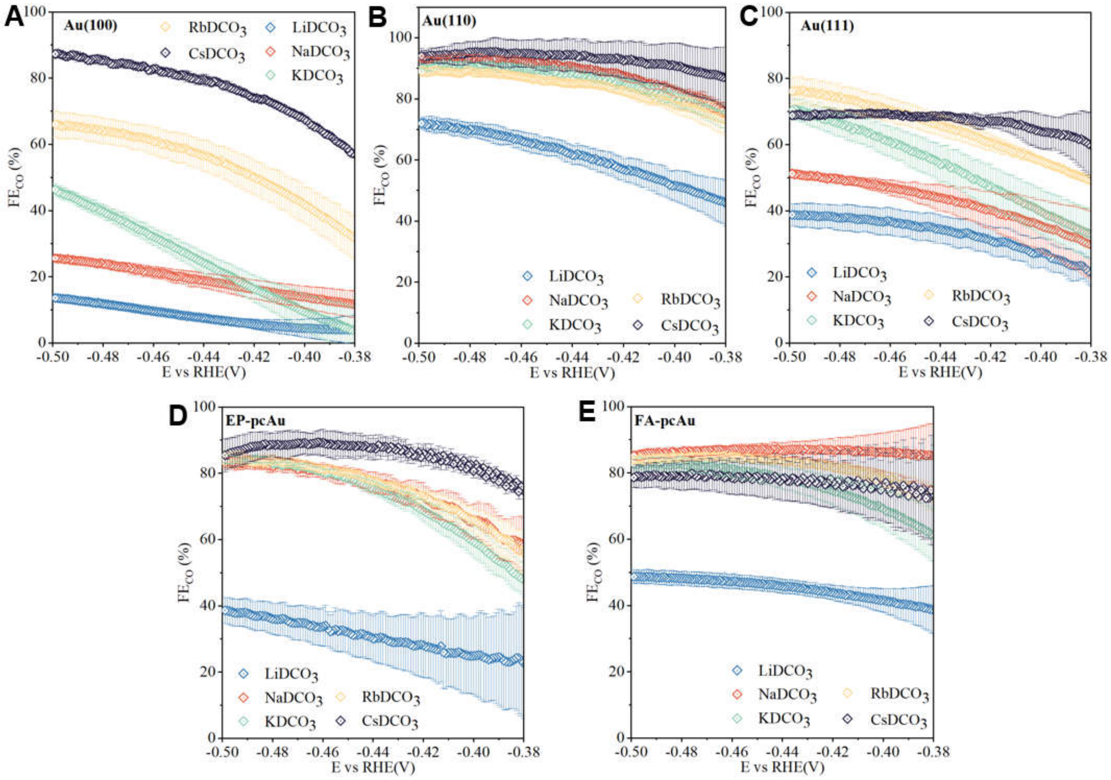

Fig. S15. Determination of the Faradic efficiencies. The Faradic efficiencies of CO calculated from RRDE voltammetry data on **(A)** Au(100), **(B)** Au(110), **(C)** Au(111), **(D)** EP-pcAu and **(E)** FA-pcAu during CO_2R at 15 mV s^{-1} and 1600 rpm in CO_2 saturated and D_2O based 0.1 M bicarbonate electrolytes (pH=6.8).

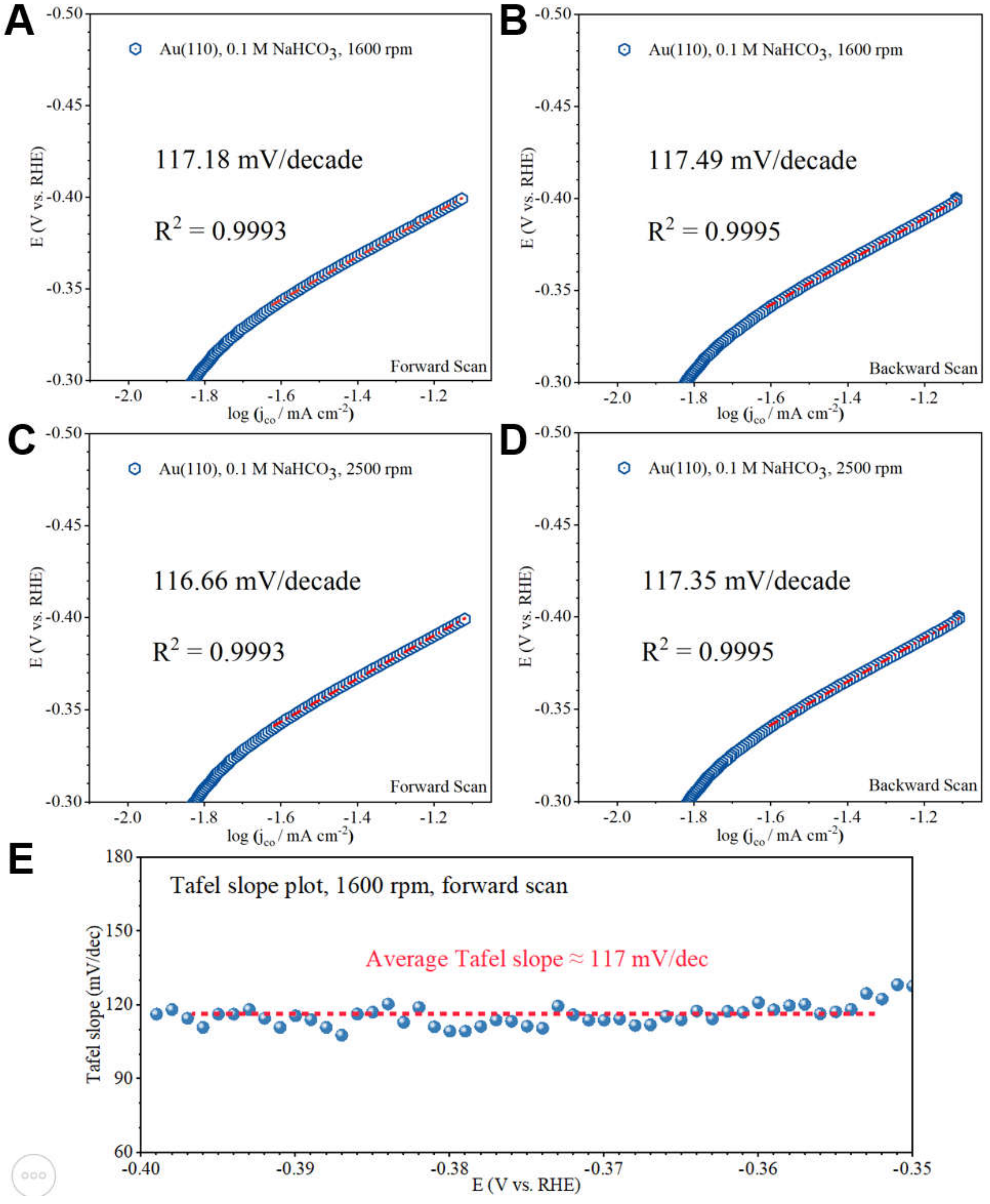

Fig. S16. Tafel analysis on Au(110). Typical Tafel plots obtained from RRDE voltammetry on Au(110) during CO_2R at 5 mV s^{-1}, **(A-B)** 1600 rpm, **(C-D)** 2500 rpm and **(E)** Tafel slope plot derived from the data in panel **(A)** (Note that Tafel slope plot was determined over a small potential range of 1 mV, this plot was used to further support the validity of the linear fitting at indicated potential region for Tafel analysis suggested by a previous study (*10*)).

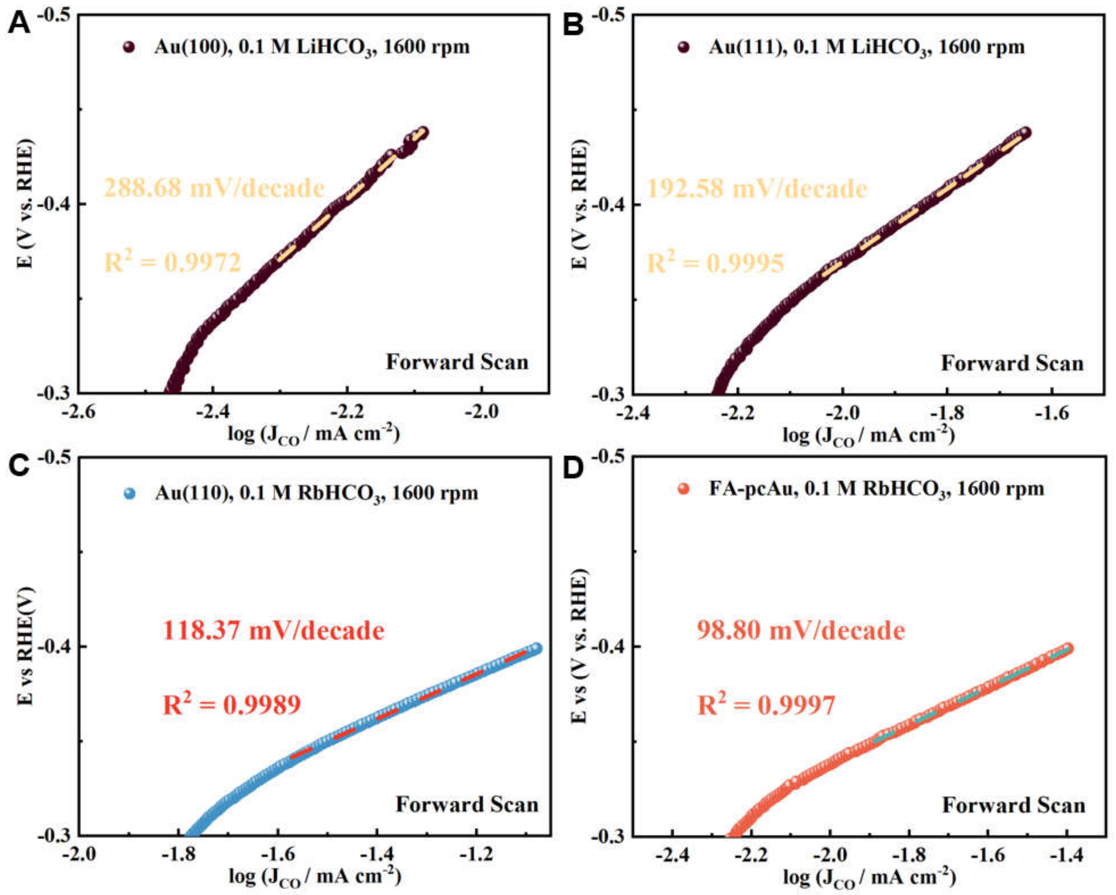

Fig. S17. Tafel analysis. The representative Tafel plots obtained from RRDE voltammetry results on **(A)** Au(100), **(B)** Au(111), **(C)** Au(110) and **(D)** FA-pcAu during CO_2R at 5 mV s^{-1}.

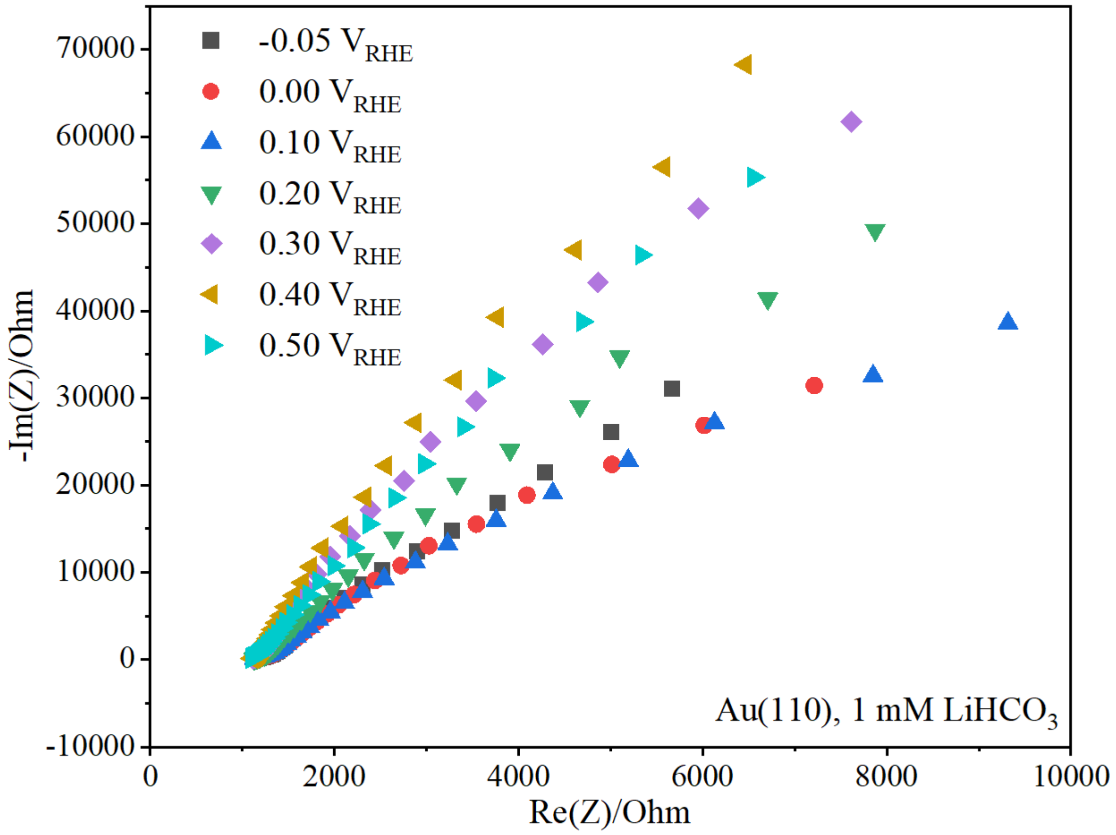

Fig. S18. Nyquist plots experiments. The representative Nyquist plots obtained on Au(110) in CO_2 saturated 1 mM $LiHCO_3$ at 1600 rpm.

Table S1. **Measured potential of zero charge values.** Potential of zero charge (PZC) values determined from PEIS results measured in various CO_2-saturated 1 mM bicarbonate electrolytes (pH=4.8).

PZC (V vs. RHE)	$LiHCO_3$	$NaHCO_3$	$KHCO_3$	$RbHCO_3$	$CsHCO_3$
Au(100)	0.32±0.02	0.33±0.05	0.33±0.02	0.13±0.02	0.45±0.04
Au(110)	0.17±0.06	0.23±0.02	0.18±0.02	0.28±0.02	0.52±0.02
Au(111)	0.52±0.02	0.45±0.04	0.45±0.04	0.10±0.04	0.17±0.02
EP-pcAu	0.15±0.04	0.25±0.04	0.17±0.02	0.12±0.02	0.52±0.02
FA-pcAu	0.15±0.00	0.07±0.02	0.27±0.05	0.23±0.02	0.38±0.02

Table S2. **Measured symmetry factor values.** The values of symmetry factor (β) fitted from Tafel slopes of CO_2R on various Au surfaces in H_2O-based 0.1 M bicarbonate electrolytes.

Symmetry factor (β)	$LiHCO_3$	$NaHCO_3$	$KHCO_3$	$RbHCO_3$	$CsHCO_3$
Au(100)	0.25±0.03	0.36±0.03	0.32±0.03	0.47±0.01	0.42±0.01
Au(110)	0.36±0.01	0.50±0.00	0.47±0.02	0.50±0.01	0.45±0.01
Au(111)	0.30±0.01	0.39±0.01	0.44±0.02	0.47±0.01	0.35±0.02
EP-pcAu	0.33±0.02	0.49±0.01	0.55±0.03	0.60±0.02	0.49±0.02
FA-pcAu	0.29±0.01	0.48±0.02	0.58±0.03	0.59±0.01	0.52±0.02

Table S3. **Measured symmetry factor values.** The values of symmetry factor fitted from Tafel slopes of CO_2R on various Au surfaces in D_2O-based 0.1 M bicarbonate electrolytes.

Symmetry factor (β)	$LiDCO_3$	$NaDCO_3$	$KDCO_3$	$RbDCO_3$	$CsDCO_3$
Au(100)	0.27±0.05	0.31±0.04	0.29±0.04	0.44±0.03	0.47±0.03
Au(110)	0.37±0.01	0.49±0.02	0.51±0.02	0.53±0.03	0.47±0.01
Au(111)	0.27±0.01	0.38±0.01	0.43±0.02	0.40±0.03	0.39±0.06
EP-pcAu	0.31±0.01	0.46±0.02	0.54±0.01	0.57±0.02	0.45±0.03
FA-pcAu	0.32±0.02	0.50±0.01	0.55±0.04	0.56±0.03	0.49±0.02

Supplementary Note 2. Analytical Grand Canonical DFT

The free energy change of concomitant adsorption and electron transfer (Eq. 1) is written as

$$\Delta G_{act}(U_{abs}) = G_{*\mathrm{CO}_2^-} - G_{\mathrm{CO}_2} - G_* + |e|U_{abs} \tag{S1}$$

where $G_{*\mathrm{CO}_2^-}$ is the free energy of the surface-bound CO_2^- species, G_{CO_2} is the free energy of CO_2 in the bulk solution, G_* is the free energy of the bare surface facet of Au, and $|e|U_{abs}$ is the free energy of the electron at a given electrode potential on the absolute scale. The potential of the electrode can be converted to the Normal Hydrogen Electrode (NHE) scale:

$$\Delta G_{act}(\mathrm{U}) = G_{*\mathrm{CO}_2^-} - G_{\mathrm{CO}_2} - G_* + |e|U - 4.6\ V \tag{S2}$$

where a typical value of 4.6 V is used and U is the electrode potential on the NHE scale (*70*).

Eq. 1 does not explicitly consider solvent molecules and cations within the electrode-electrolyte interface that can be involved in this reaction step. The most simplified DFT model based on Eq. S2 presumes an integer electron transfer during CO_2 adsorption, an assumed symmetry factor of $\beta = 1$. To further incorporate electrochemical double layer (EDL) considerations and examine the variation of β with EDL properties, we assign a reference activation barrier (ΔG_{act}^0) calculated for neutral systems to the potential of zero charge of the bare surface:

$$\Delta G_{act}^0(\mathrm{U}) = G_{*\mathrm{CO}_2^-} - G_{\mathrm{CO}_2} - G_* + |e|U_{pzc} \tag{S3}$$

$$U_{pzc} = \left(\frac{\emptyset_{bare} - 4.6\ V}{e}\right) \tag{S4}$$

where $\emptyset_{bare}$ is the work-function of the bare metal surface. The Gibbs free energy of any species, λ is made dependent on the electrode potential and interfacial electric field within the EDL:

$$G_\lambda(U) = G_\lambda\left(U_{PZC,\lambda}\right) - \frac{1}{2}C_{dl}\left(U - U_{PZC,\lambda}\right)^2 + \mu_\lambda F_\lambda + \alpha_\lambda \frac{1}{2}{F_\lambda}^2 \qquad (S5)$$

The first term is $G_\lambda\left(U_{PZC,\lambda}\right)$, the free energy of species λ at the U_{pzc} of species λ. The second term is the energy required to charge the surface to U from $U_{PZC,\lambda}$ as dependent on the capacitance of the double layer, C_{dl}. The last two terms are the interaction between the adsorbate with the electric field F_λ dictated by the species surface-normal dipole moment (μ_λ) and polarizability (α_λ). The values of $G_\lambda\left(U_{PZC,\lambda}\right)$, $U_{PZC,\lambda}$, and μ_λ are all computed in a standard optimization of the species (bare surface or surface with adsorbate). The value of α_λ is computed by measuring the response of the system to an applied electric field along the surface normal direction. Details of computing α_λ are discussed by Agrawal et al (*39*). The capacitance and the potential dependence of the electric field are approximated using a theoretical model of the EDL. In this work, we used a Helmholtz model to approximate the ion distribution within the EDL, expressing the capacitance and the electric field as:

$$C_{dl} = \frac{\varepsilon A}{d} = \frac{\varepsilon_r \varepsilon_0 A}{d} \qquad (S6)$$

$$F_\lambda = \frac{U - U_{pzc,\lambda}}{d} = \frac{U - \left(U_{pzc} + \frac{\mu_\lambda}{\varepsilon_r \varepsilon_0 A}\right)}{d} \qquad (S7)$$

where ε_r is the relative permittivity of the media within the EDL, ε_0 is the vacuum permittivity, d is the width of the EDL described by a parallel plate description of the countercharge of ions, and A is the area of the metal surface. The U_{pzc} of any reaction state λ, $U_{PZC,\lambda}$, can be determined from the dipole moment of reaction state λ relative to U_{pzc} of the bare surface. An advantage of using a Helmholtz model is the ability to quantify the sensitivity of model predictions based on approximations of the physical properties of the EDL (d and ε_r). Combining equations S3-S7 gives the potential dependent activation barrier:

$$\Delta G_{act}(U') = \Delta G^{o}_{act} + eU' - \frac{1}{2}\frac{\Delta(\mu^2)}{\varepsilon A d} + \frac{\Delta\mu}{d}U' - \frac{\Delta(\mu^2)}{\varepsilon A d} + \frac{\Delta\mu}{d}U' + \frac{\Delta(\alpha\mu^2)}{2\varepsilon^2 A^2 d^2} - \frac{\Delta(\alpha\mu)}{\varepsilon A d^2}U' + \frac{1}{2}\frac{\Delta\alpha}{d^2}U'^2 \quad (S8)$$

$$U' = U - U_{pzc} \quad (S9)$$

Additional details of the derivation and implications of this analytic Helmholtz model for approximating electrochemical activation barriers can be found in Agrawal et al (*39*).

The symmetry factor is calculated by taking the first derivative of the activation barrier w.r.t the applied potential as noted in Eq. 3. For a reaction consisting of the movement of a positively charged species from the bulk to the surface, we obtain the symmetry factor in the form of Eq. 3. If reaction does not involve moving a positively charged species from the bulk electrolyte to the surface, such as when adsorbing CO_2, $|e|$ does not appear in equation S10, and we rewrite the equation as:

$$\beta|e|(U) = \frac{\partial \Delta G_{act}(U)}{\partial U} = 2\frac{\Delta\mu}{d} - \frac{\Delta(\alpha\mu)}{\varepsilon A d^2} + \frac{\Delta\alpha}{d^2}(U - U_{pzc}) \quad (S10)$$

For such a reaction, β takes on a non-zero value because the dipole moment (and polarizability) change upon reaction. This would occur if CO_2 took on a negatively charged state when adsorbed, leaving a positive charge in the metal surface for the neutral system and creating a significant dipole moment relative to the bare surface model. Both equations 3 and S11 allow us to quantify how the symmetry factor is sensitive to both the electronic character of the transition state and the approximation of the electrode-electrolyte interfacial properties (ε and d for the Helmholtz model used herein).

Supplementary Note 2.1 Considerations when calculating the symmetry factor

For the simplest case, the calculation of the dipole moment and polarizability changes in Eq. S1 of $*CO_2^-$ are relative to CO_2 in the gas-phase and the bare metal

surface. For the case of the explicit coordinated cation-CO_2 complex with explicit H_2O, the reaction is written as:

$$\mathrm{CO_2} + * + \mathrm{e^-} + Cat^+ + nH_2\mathrm{O} \rightarrow * CO_2(\mathrm{Cat})(H_2\mathrm{O})_n \qquad (S11)$$

where gas-phase CO_2, the cation denoted as Cat^+, and n number of H_2O molecules undergo a surface reaction to form the cation coordinated CO_2 complex. The dipole moment and polarizability change are referenced to the bare Au surface facet, gas-phase CO_2, gas-phase K^+, and H_2O molecules when applicable to the explicit cation models. In practice, the dipole moment and polarizability changes are referenced similarly as the case without explicit consideration of the alkali cation and/or the H_2O as the bare metal surface dipole moment is the reference state.

For the formation of the co-adsorbed model of the $*CO_2$ and the surface-bound alkali cation, this reaction is shown as:

$$\mathrm{CO_2} + \ 2 * + 2\,\mathrm{e^-} + Cat^+ + nH_2\mathrm{O} \rightarrow * \mathrm{CO_2^-} + * \,(\mathrm{Cat})(H_2\mathrm{O})_n \qquad (S12)$$

where the dipole moment and polarizability changes are referenced identically as the first case.

Supplementary Note 2.2 Considerations when calculating the symmetry factor

Before examining the properties of the specific CO_2 reduction species, we examine the functional dependence of the symmetry factor on ε_r and d. The effective width of the EDL is typically unknown for any given system, though we can calculate β for a plausible range of d. **Fig. S19** plots the symmetry factor as a function of d for different reaction dipole moment changes, using an interfacial dielectric constant of 2 (a reasonable first approximation for a water region close to a metal surface) (*40, 41*).

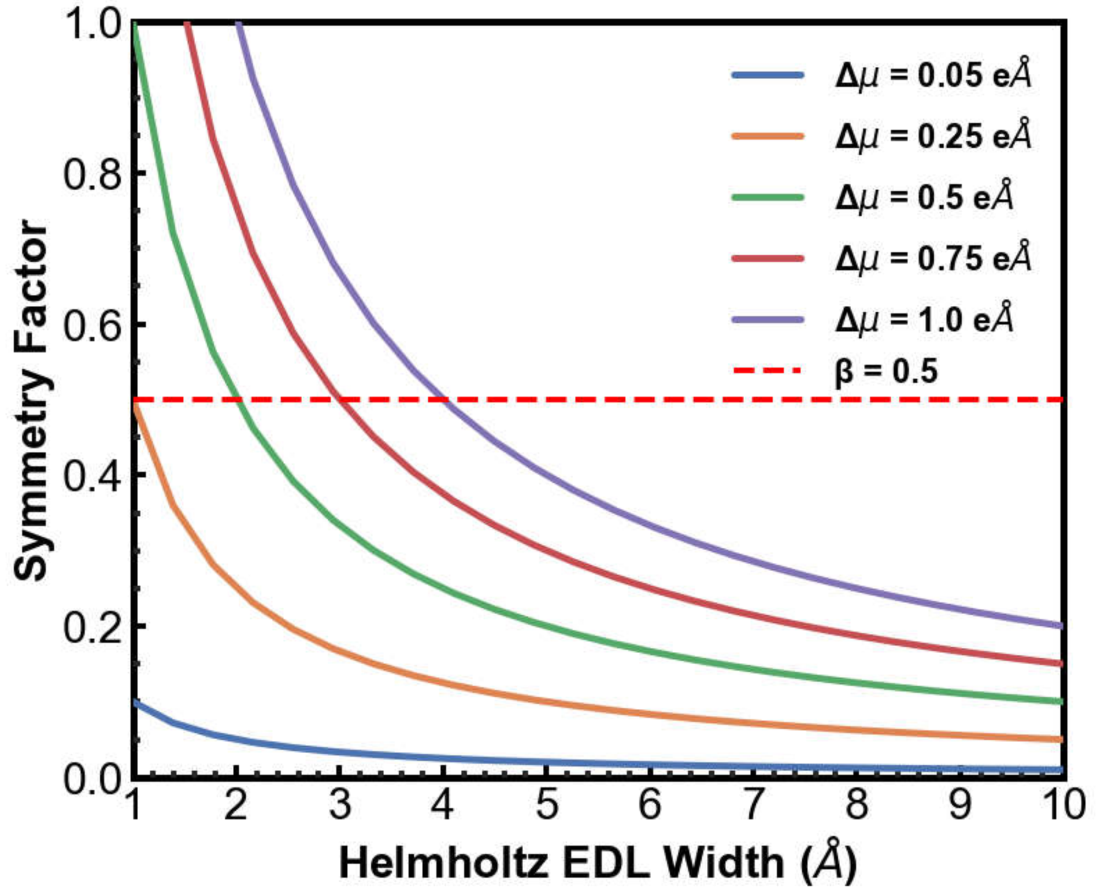

Fig. S19. Calculated β as a function of EDL width. Profile of β as both the Helmholtz EDL width d and reaction dipole moment change is varied, as calculated with Equation 12b. A dashed line is included for $\beta = 0.5$ as a reference. The reaction polarizability change was set to 0. A dielectric constant of 2 was used. The area of the Au (111) surface was used.

The magnitude of β is strongly dependent on the reaction dipole moment change. Given an arbitrary EDL width of 3 Å, predicted values of β span from 0.03 to 0.68 for respective dipole moment changes from 0.05 e^- Å to 1.0 e^- Å. The Helmholtz EDL width (d) also strongly impacts the magnitude of β. For $\beta = 0.5$, a smaller EDL width (countercharges close to the surface) would be needed for reaction pathways with smaller dipole moment changes. For $\Delta\mu = 0.25$ e^-Å to 1.0 e^-Å, the predicted EDL width ranges from 1 Å to 4 Å to match a $\beta = 0.5$. This highlights that DFT predicted β are

sensitive to the magnitude of the dipole moment change upon reaction and how close the countercharges are to the surface.

Other properties that can potentially affect the magnitude of β are the polarizability change ($\Delta\alpha$ and $\Delta\alpha\mu$) and the dielectric constant of the medium within the EDL. The dependence of β on these properties are discussed in **Supplementary Note 2.** Polarizability changes along the reaction path can impact β, however, β is relatively invariant to the choice of the dielectric constant. The dependence of β on dipole moment change for a reaction involving transferring a cation (i.e., when Eq. 3 is applicable) is shown in **Fig. S20**.

When calculating the symmetry factor, the incorporation of the polarizability change along the reaction pathway introduces a potential independent and dependent term.

$$\beta|e|(U) = 2\frac{\Delta\mu}{d} - \frac{\Delta(\alpha\mu)}{\varepsilon A d^2} + \frac{\Delta\alpha}{d^2}(U - U_{pzc}) \qquad (S13)$$

Contributions from this potential dependent term were considered as an average contribution over an arbitrary potential range from -1 V_{NHE} to 1 V_{NHE}. The sensitivity of β based on the magnitude of the polarizability change was investigated in **Fig. S18**.

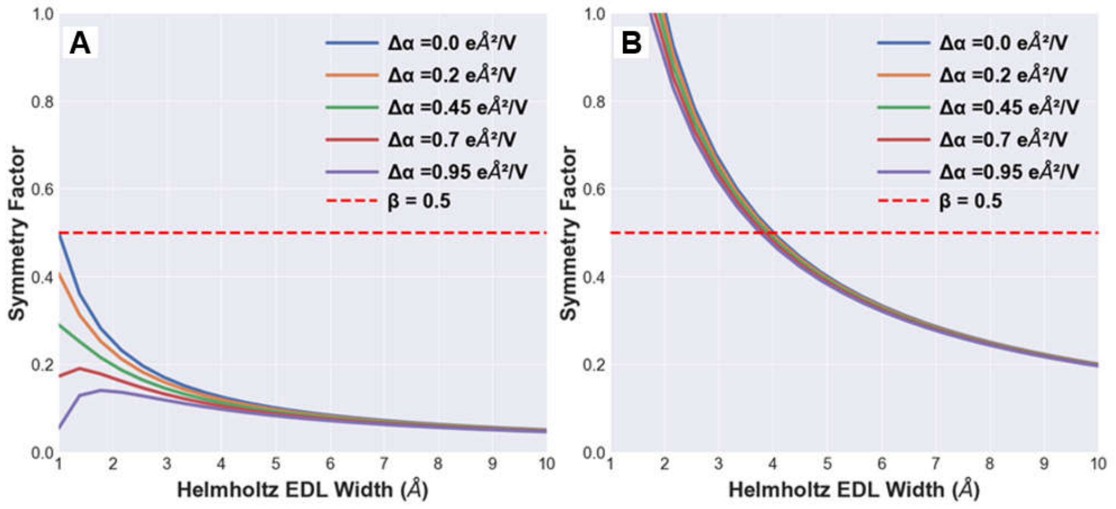

Fig. S20. Sensitivity analysis. Sensitivity of the symmetry factor based on the magnitude of the polarizability change for a dipole moment change of **(A)** 0.25 e^{-}Å and **(B)** 1.0 e^{-}Å. A value of 0.5 for β is given by a red dashed line for reference. Arbitrarily range of magnitude of polarizability changes were used. A dielectric constant of 2 was used. The area of Au (111) surface was used.

Fig. S20A illustrates the case of a reaction path model with a "smaller" dipole moment change of $\Delta\mu$ = 0.25 e^{-}Å. Given an EDL width of 3 Å, β reduces from 0.17 to 0.12 for $\Delta\alpha$ = 0 to 0.95 e^{-} Å^{2}V^{-1}. If the EDL was smaller such as d= 1.5 Å, β can vary more significantly as β decreases 0.28 to 0.14 for $\Delta\alpha$ = 0 to 0.95 e^{-} Å^{2}V^{-1}. **Fig. S20B**

considers a reaction path model with a much larger dipole moment of $\Delta\mu$ = 1.0 e$^-$Å. Calculated values of symmetry factor show minimal variations with varying magnitude of the polarizability change along the reaction path with a large dipole moment change.

A profile of β for a system with a faradic electron transfer is shown w.r.t the Helmholtz EDL width in **Fig. S21**. Keeping the EDL width constant, larger dipole moment changes results in deviations away from one and converges towards zero as seen in **Fig. S21A**. Similar to reaction paths with positive dipole moment changes in **Fig. 6**, the polarizability change has a minor contribution to the magnitude of beta as shown in **Fig. S21B**.

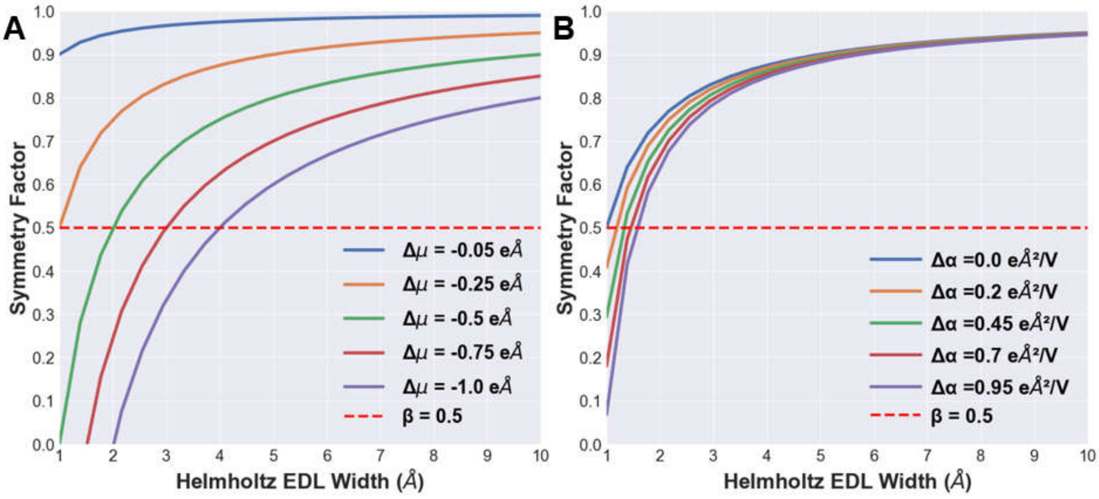

Fig. S21. Sensitivity analysis. (A) Profile of β as the both the Helmholtz EDL width and dipole moment change is varied. A value of 0.5 was arbitrarily chosen for β. Arbitrarily range of magnitude of dipole moment changes were used. No polarizability change was considered. A dielectric constant of 2 was used. The area of Au (111) surface was used. **(B)** Profile of β as the both the Helmholtz EDL width and polarizability change is varied. A constant dipole moment change of $\Delta\mu$ = -0.25 e$^-$Å was kept constant. A value of 0.5 was arbitrarily chosen for β. Arbitrarily range of magnitude of polarizability changes were used.

Fig. S22 shows that the profile of β_{DFT} is invariant to the choice (**Fig. S20A**

and **S20B**) of the dielectric constant until a significantly large polarizability is used (**Fig. S20C**). As shown previously, β_{DFT} is mainly sensitive to the EDL width and the magnitude of the dipole moment change along the reaction path.

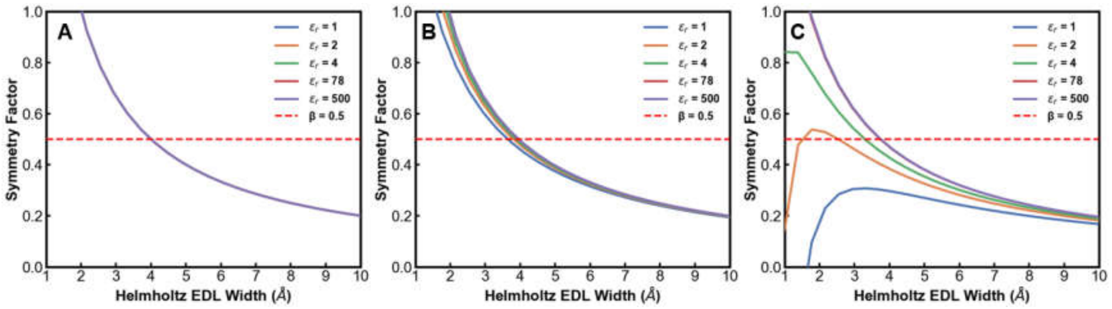

Fig. S22. A comparison of β_{DFT} with varying EDL width and dielectric constant. Profile of β_{DFT} as both the EDL width and dielectric constants are varied at polarizability changes of a) 0.25 $e^-Å^2/V$, b) 0.5 $e^-Å^2/V$, and c) 1 $e^-Å^2/V$. A dipole moment change of 1 e^- Å is used. The area of Au (111) surface was used.

Supplementary Note 3. Simplest model for the formation of $*CO_2^-$ on Au surface facets

Concomitant electron transfer and adsorption of CO_2 was first modeled with no explicit H_2O molecules or alkali metal cations. **Fig. S23** shows the proposed reaction path of this reaction step and comparison between DFT predicted values of β and experimentally observed (**Fig. 5**) values of β.

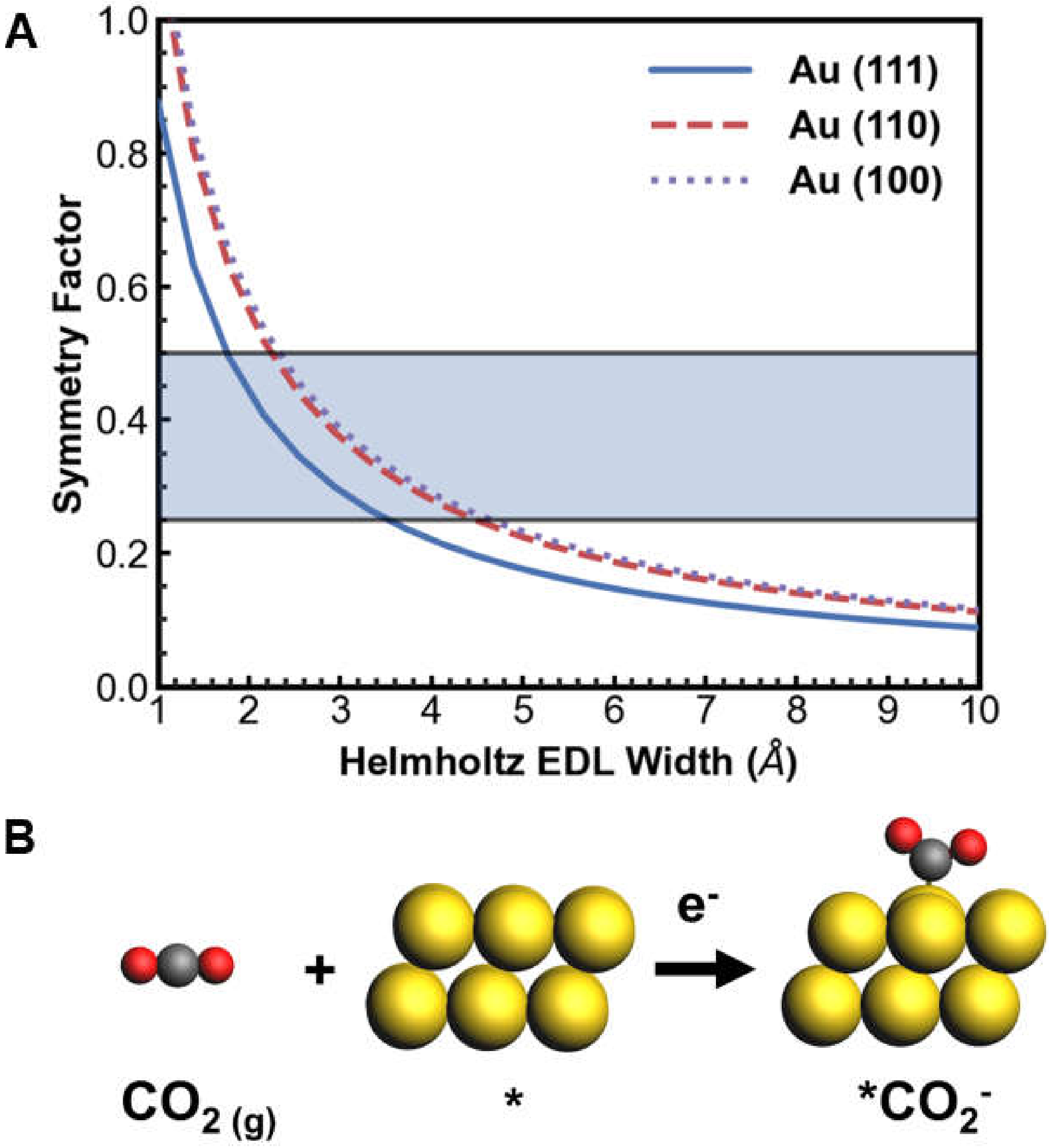

Fig. S23. DFT model for the formation of $*CO_2^-$ on Au surface facets. (A) DFT predicted symmetry factors are plotted versus the presumed EDL width for the non-solvated electron transfer upon adsorption of CO_2. Predicted β values are plotted for Au

(111) (blue solid line), Au (110) (red dashed line), and Au (100) (purple dotted line). A dielectric constant of 2 is used for calculating β_{DFT}. The blue shaded region indicates the range of experimentally observed values of β_{exp} = 0.25 to 0.5. **(B)** DFT geometries of non-solvated CO_2 electron transfer. Atom colors in b are as follows: yellow = Au, grey = C, and red = O.

The geometry of the $*CO_2^-$ species was obtained by removing the H atom from an adsorbed COOH* species and performing a constrained optimization fixing the CO_2 position. While this does not guarantee a local minima structure of $*CO_2^-$, it fixes the molecule in a bent configuration representative of $*CO_2^-$ for determination of the surface normal dipole moment. The dipole moment changes for the formation of $*CO_2^-$ are similar across facets, ranging from 0.48 e^- Å to 0.59 e^- Å for Au (111) and Au (100) respectively. If the EDL width is constant across facets of Au, DFT predicted values of β would increase from the (111) < (110) < (100) facet.

Fig. S23 infers that values of *d* would need to range from 1.8 Å to 4.5 Å to match experimental values of β. Analysis of the charge density of an excess electron across different surface facets of Au suggests that the charge plane in the metal is predicted to be 0.8 to 1.1 Å above the nuclei of the metal, as shown in **Fig. S24**. This suggests that the actual countercharge plane is located a minimum 2.6 Å and maximum 5.5 Å from the metal nuclei plane. The distance between O of $*CO_2^-$ and this same nucleus plane is approximately 2.9 Å, leaving a maximum gap distance between O and the cation countercharge plane of 2.6 Å. A 2.6 Å countercharge distance is unphysical as it would imply the countercharge cations approach the surface closer than O of $*CO_2^-$ and closer than a typical metal-cation bond distance (Table **S4**).

Table S4. **Bond distances of specifically adsorbed cations on Au (111), (110), and (100) surface facets.** The hollow adsorption site was considered across all surface facets.

Cation-Au Bond Distance (Å)			
**Cation**	**Au (111)**	**Au (110)**	**Au (100)**
Li^+	2.62	2.78	2.68
Na^+	2.96	3.20	2.96
K^+	3.24	3.38	3.34
Rb^+	3.36	3.49	3.47
Cs^+	3.50	3.61	3.59

Even with the maximum metal-countercharge distance of 5.5 Å, the resulting gap of 2.6 Å between $*CO_2^-$ and the counterchange distribution would not leave room for any water between the adsorbed CO_2 and the countercharge (*41, 44*). This infers that the cation must be above the $*CO_2^-$ to where it can potentially undergo a coordinative interaction. We conclude that representing this CO_2 adsorption/electron transfer step without direct interactions of the $*CO_2$ with water and cations is not consistent with experimentally observed values of β.

Dipole moments and polarizability of each state involved in the formation of $*CO_2^-$ are given in Table **S5**.

Table S5. Dipole moment and polarizability of *CO_2^- on Au (111), Au (110), and Au (100) surfaces.

Surface Facet	Dipole Moment (e^-Å)	Polarizability (e^-Å^{2}V^{-1})
Au(100)	0.59	4.92
Au(110)	0.57	5.20
Au(111)	0.48	4.76

A minimum dipole moment change of 0.74 e^- Å is required to match the maximum β_{exp}= 0.5 at EDL width of 2.9 Å (distance between O of *CO_2^-). Dipole moment changes of *CO_2^- on Au (111), Au (110), and Au (100) surface are too small as they range from $\Delta\mu$ = 0.44 to 0.58 e^- Å. Adsorbed CO_2, without solvation or explicit interactions with cations, does not take on sufficient negative charge to be consistent with the symmetry factors observed experimentally.

A different reaction path model is required to represent the electro-kinetics of *CO_2^- formation. **Section 3.2.1** explores the involvement of explicit cations and water molecules. Before considering explicit solvent/cation involvement, it is also possible a larger dipole moment could result simply from the *CO_2 formation transition state forming further from the surface. CO_2^- could be formed in the solution phase (*71*), which could also potentially increase the $\Delta\mu$ for *CO_2^- formation as the distance of *CO_2^- away from the surface increases. This was investigated in **Supplementary Note 4**, where the formation of CO_2^- in the solution phase is unlikely as CO_2^- would reside

unphysically past the EDL region or too close to the surface in order to produce large enough dipole moment changes to represent β_{exp}.

The distance where the excess electron (electron spillover) resides above the top surface atom across different surfaces facets of Au was determined in **Fig. S24**.

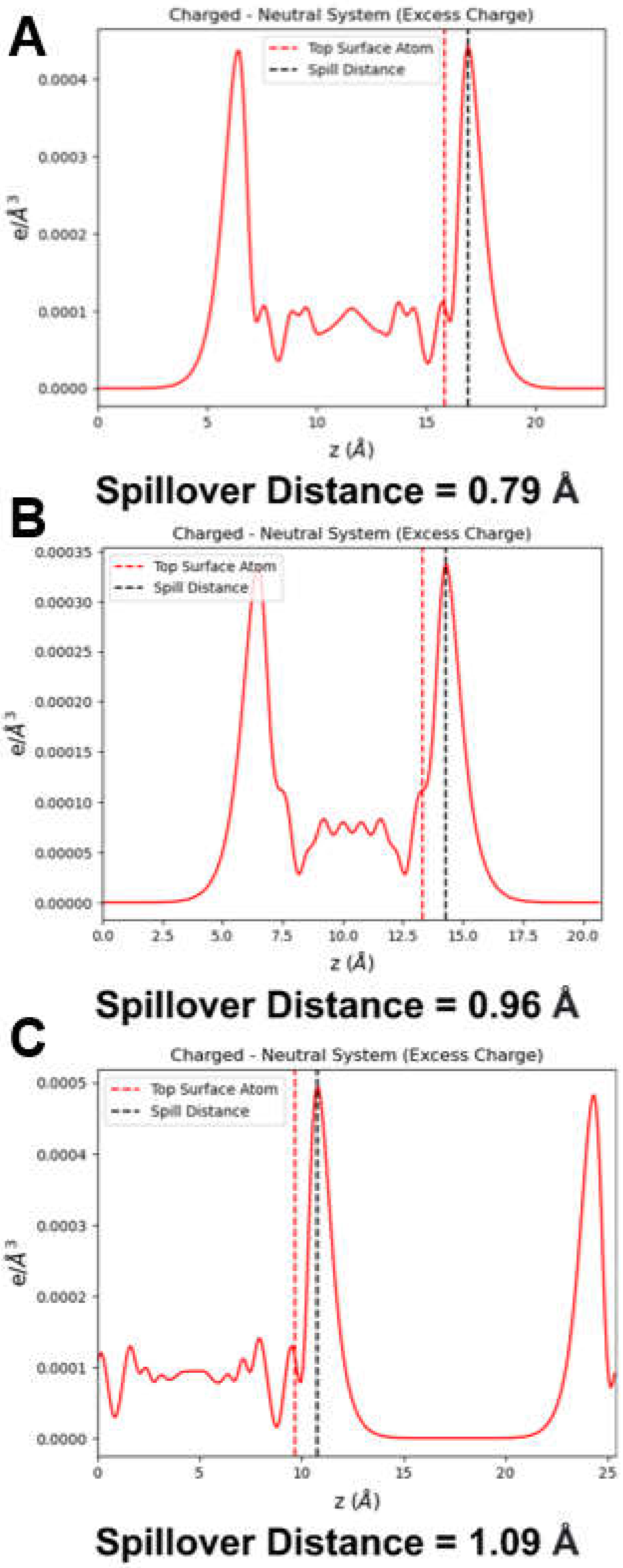

Fig. S24. Determination of electron spillover distances. Electron spillover distances on **(A)** Au (111), **(B)** Au (110), and **(C)** Au (100) surface facets. Charge density is shown as the difference of average charge per volume basis between the Au surface facet with an excess electron and a neutral surface facet of Au.

The excess electron was determined by taking the difference between the charge density of an Au surface facet with an excess electron and the neutrally charged surface. The charge density is shown as the average charge per volume basis. The spillover distance across different facets varies from 0.79 Å to 1.09 Å. suggesting that the electron does not reside at the nuclei of the surface metal atom but above the Au surface atom.

Supplementary Note 4. Investigation of formation of CO_2^- in the solution phase

Discussion in **Section 3.2.1** suggests that a simple surface-bound $*CO_2^-$ model is an inadequate model of the reaction path due to the insignificant dipole moment change upon formation at the surface. The dipole moment change of CO_2^- formation can be increased if CO_2^- is formed farther away from the surface and is stable. Solution phase reduction potentials suggested that the formation of CO_2^- is possible at significant overpotentials (*71*). This increase in dipole moment change can then lead to larger values of the symmetry factor that can match β_{exp} as shown in **Fig. S25A**. This increase in dipole moment change can be demonstrated by displacing the $*CO_2^-$ farther from the surface (increasing the separation of charge) while maintaining the bent O-C-O structure of the surface-bound state (constrained geometry). **Fig. S25B** shows how the dipole moment change of CO_2^- formation increases as the distance between the nucleis of the Au surface atom and the C of CO_2^- ($d_{Au\text{-}C}$) as the black dashed line.

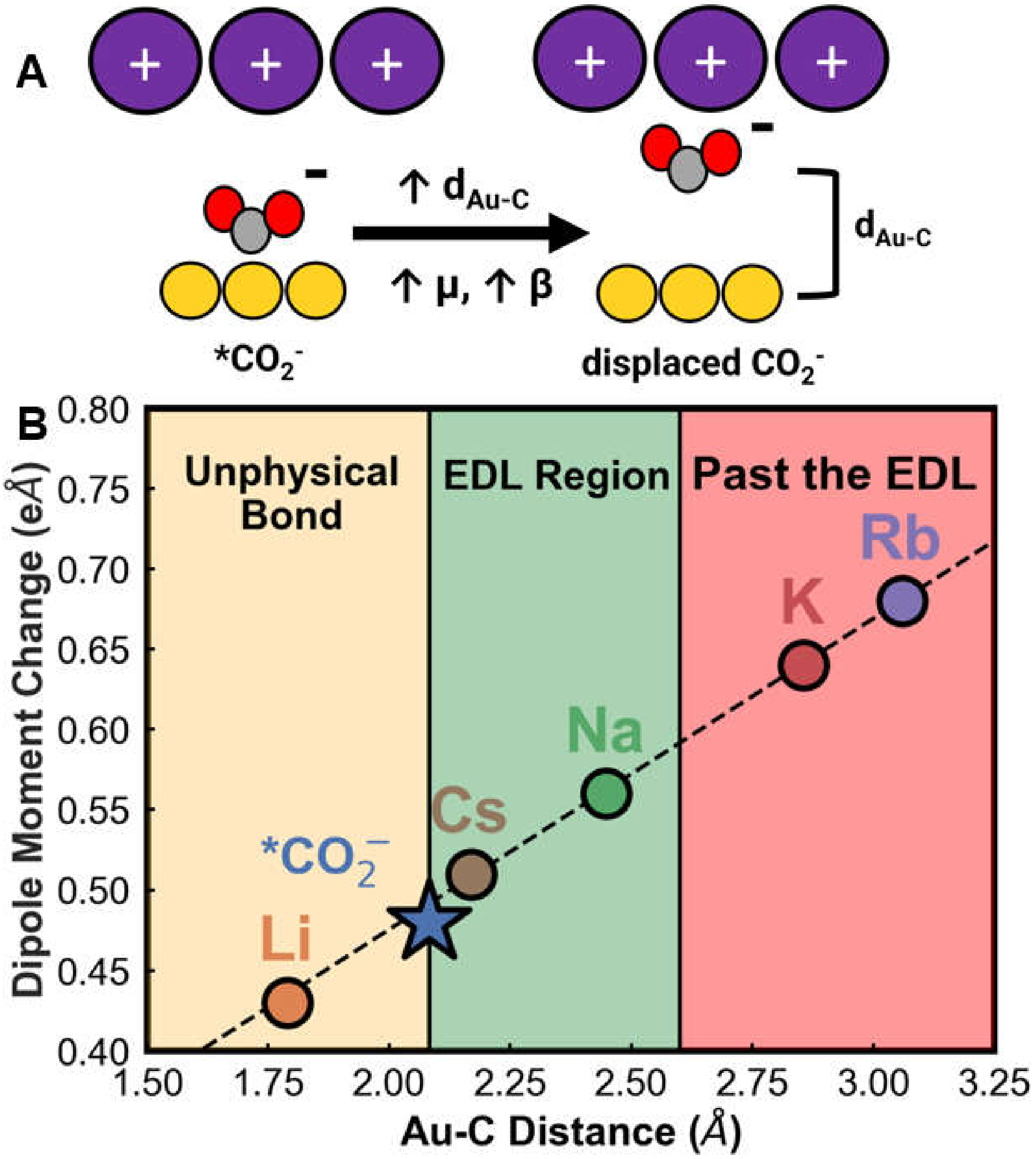

Fig. S25. Investigation of formation of CO_2^-. (A) Graphical representation of displacing surface bound $*CO_2^-$ into the solution phase could result in larger dipole moment changes **(B)** Increase of the dipole moment of CO_2^- formation as the CO_2^- is displaced farther from the surface. The blue star marker is the dipole moment change corresponding to the formation of surface-bound $*CO_2^-$. Black dashed line is a linear fit of dipole moment changes of CO_2^- at different $d_{Au\text{-}C}$ obtained from single-point geometry optimizations, extrapolating from $d_{Au\text{-}C}$ beyond from the DFT predicted bond length of surface-bound $*CO_2^-$. The circle-colored markers are the dipole moment changes required from β_{exp} for different electrolyte compositions assuming an Helhomltz EDL width of 2.6 Å. Using the dipole moment changes inferred β_{exp}, $d_{Au\text{-}C}$

for different electrolyte compositions can be calculated using the linear regression line determined from DFT. The yellow shaded region corresponds to Au-C bond lengths smaller than DFT predicted bond lengths based on the blue star marker. The green shaded region corresponds to CO_2^- within the EDL region based on a predicted countercharge distance of 2.6 Å as discussed in Supplementary Note 3. The red shaded region represents the unphysical region where CO_2^- resides beyond the countercharge distribution and outside of the EDL.

Fig. S25B shows that surface-bound $*CO_2^-$ Au-C bond length is 2.1 Å on the Au (111) surface. The yellow region represents unphysical approach of CO_2^- for $d_{Au\text{-}C}$ < 2.1 Å. The green region represents the EDL region defined by a Helmholtz countercharge of 2.6Å away from the surface where physically CO_2^- could reside as either surface-bound or displaced in solution ($2.1 \leq d_{Au\text{-}C} < 2.6$ Å). The red region represents an unphysical region where CO_2^- resides within or past the Helmholtz countercharges ($d_{Au\text{-}C} > 2.6$ Å).

Given the β_{exp} from **Fig. 5** for different cation identities, the corresponding dipole moment changes and distances of displacement required of CO_2^- can be determined using the DFT determined linear fit (black dashed line). The dipole moment changes to form the surface-bound $*CO_2^-$ is too small w.r.t Na^+, K^+, Rb^+, and Cs^+. The dipole moment of $*CO_2^-$ is too large to match β_{exp} for Li^+. A smaller dipole moment change can be obtained by reducing the separation of charge between CO_2^- and Au but results in unphysical bond lengths according to DFT (yellow region defined by $d_{Au\text{-}C} <$ 2.1 Å). In order to match β_{exp} for Na^+, K^+, Rb^+, and Cs^+ composing electrolytes, the separation of charge needs to be increased (increasing $d_{Au\text{-}C}$) to represent large enough dipole moment changes. Rb^+ and K^+ shows that the displacement of CO_2^- from the Au (111) has to be so large that the CO_2^- would unphysically reside past the Helmholtz EDL countercharge (yellow region defined by $d_{Au\text{-}C} <$ 2.6 Å). While Na^+ and Cs^+ is able

to reside within the EDL region based on our current model, we cannot determine the stability of CO_2^- at these values of d_{Au-C}, whether CO_2^- preferentially would adsorb the surface or maintain the same bent structure and angles as the $*CO_2^-$ (a key assumption in this analysis). We extend this analysis for the Au (100) and Au (110) surfaces (Figure S24 and approach similar conclusions that solution phase formation of CO_2^- is unlikely.

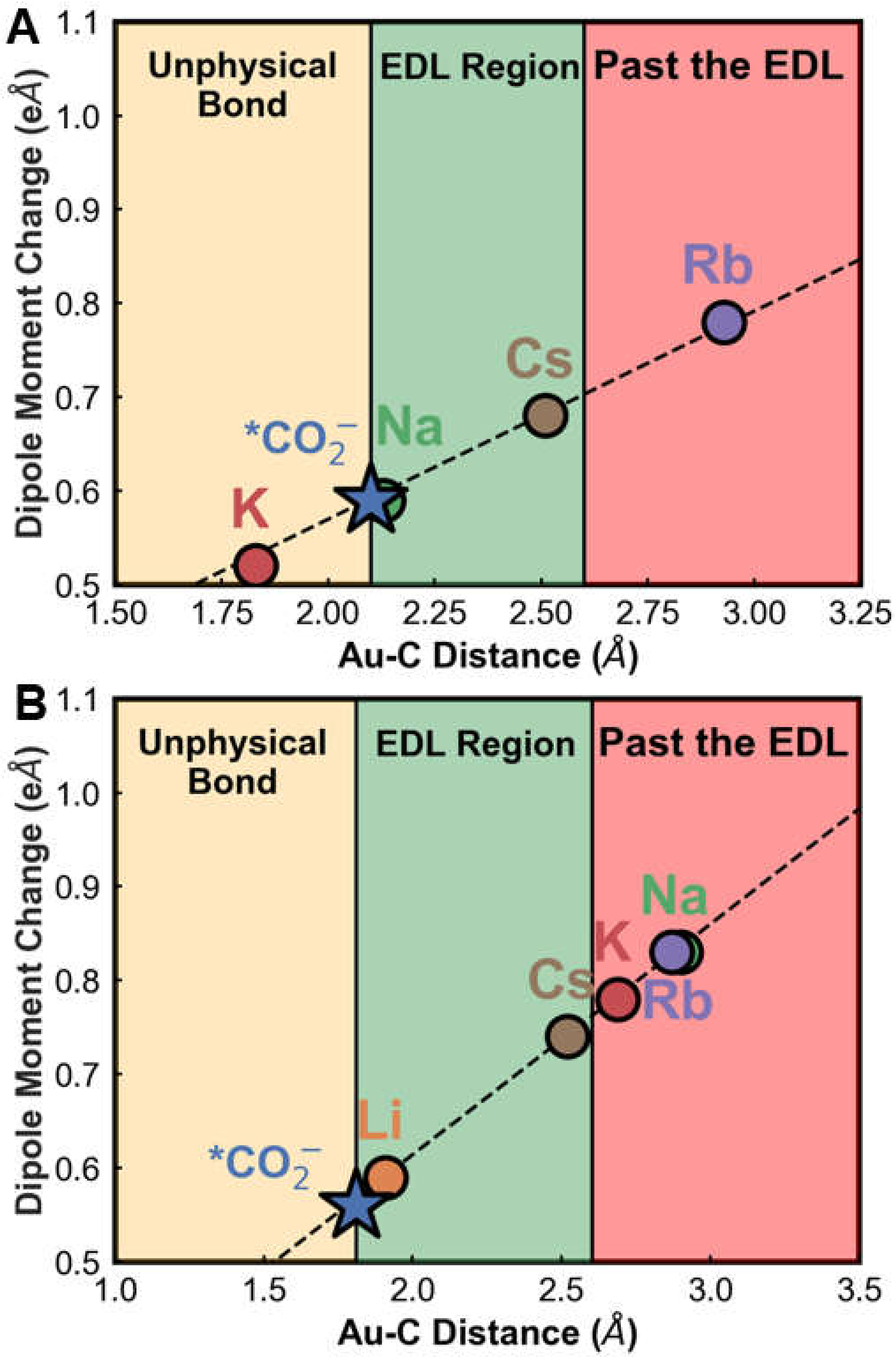

Fig. S26. Dipole moment change as a function of Au-C distance. Increase of the dipole moment of CO_2^- formation as the CO_2^- is displaced farther from the **(A)** Au (100) and **(B)** Au (111) surface, the blue star marker is the dipole moment change corresponding to the formation of surface-bound $*CO_2^-$. Black dashed line is a linear

fit of dipole moment changes of CO_2^- at different $d_{Au\text{-}C}$ obtained from single-point geometry optimizations for the Au (100) and Au (110) surface, extrapolating from $d_{Au\text{-}C}$ beyond from the DFT predicted bond length of surface-bound $^*CO_2^-$. The circle-colored markers are the dipole moment changes required from β_{exp} for different electrolyte compositions assuming an Helhomltz EDL width of 2.6 Å. Using the dipole moment changes inferred β_{exp}, $d_{Au\text{-}C}$ for different electrolyte compositions can be calculated using the linear regression line determined from DFT. The yellow shaded region corresponds to Au-C bond lengths smaller than DFT predicted bond lengths based on the blue star marker. The green shaded region corresponds to CO_2^- within the EDL region based on a predicted countercharge distance of 2.6 Å as discussed in **Supplementary Note 3**. The red shaded region represents the unphysical region where CO_2^- resides beyond the countercharge distribution and outside of the EDL.

Supplementary Note 5. Formation of $*CO_2^-$ with explicit cations and H_2O

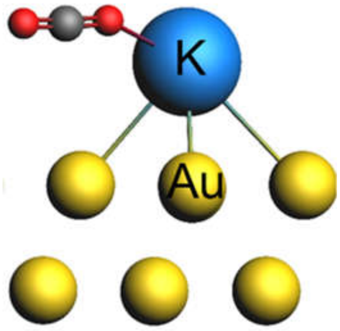

Fig. S27. Optimized geometry of linear $*CO_2$ with co-adsorbed K* on Au (111) surface. Atom colors are as follows: Blue = K, Yellow = Au, Grey = C, and Red = O.

Table **S6** shows that the dipole moment and polarizability vary across reaction models of the transition state for $*CO_2^-$ on Au (111) surface in K^+ electrolyte. Table **S6** clearly shows that only the coordinated cation model produces physically reasonable EDL width that could occurring during the formation of $*CO_2^-$.

Table S6. Dipole moment and polarizability of *CO_2^- and *$K(H_2O)_n$ coordinated and co-adsorbed transition state on Au (111) surface. Dipole moment and polarizability changes are relative to the bare metal surface.

Transition State	Dipole Moment (e^-Å)	Polarizability ($e^-Å^2V^{-1}$)	EDL Width at $\beta_{DFT} = \beta_{exp}$ (Å)
*CO_2K	-1.60	5.53	5.9
*$CO_2K(H_2O)$	-1.60	6.83	5.8
*CO_2^- + *$K(H_2O)$	-0.56	5.03	2.0
*CO_2^- + *$K(H_2O)_2$	-0.47	5.15	1.6
*CO_2^- + *$K(H_2O)_3$	-0.35	5.12	1.3
*CO_2^- + *$K(H_2O)_4$	-0.45	5.30	1.5
*CO_2^- + *$K(H_2O)_5$	-0.44	5.43	1.5

Table **S7** shows sensitivity of dipole moments and polarizability of the co-adsorbed *CO_2^- and *(Cation)$(H_2O)_n$ models w.r.t the number of explicit H_2O included. *CO_2^- is only stabilized by a cation coordinate model for Li^+ without explicit H_2O. Changes in dipole moment are primarily due to the orientation and number of waters considered explicitly. Despite this variation, the co-adsorbed explicit cation model of *CO_2^- is a poor reaction model due to its insufficient dipole moment changes.

Table S7. EDL widths predicted from cation co-adsorbed *CO_2^- reaction path models at $\beta_{DFT} = \beta_{exp}$. Dipole moment and polarizability of *CO_2^- and *(X)(H_2O)n co-adsorbed transition state on Au (111) surface for X= Li^+, Na^+, Rb^+, and Cs^+. Dipole moment and polarizability changes are relative to the bare metal surface.

Cation	Transition State	Dipole Moment (e^-Å)	Polarizability (e^-Å^{2}V^{-1})	EDL Width at $\beta_{DFT} = \beta_{exp}$ (Å)
Na^+	*CO_2^- + *Na(H_2O)	-0.61	4.89	1.9
	*CO_2^- + *Na(H_2O)$_2$	-0.44	5.04	1.1
	*CO_2^- + *Na(H_2O)$_3$	-0.34	5.08	1.0
	*CO_2^- + *Na(H_2O)$_4$	-0.50	5.15	1.6
	*CO_2^- + *Na(H_2O)$_5$	-0.50	5.22	1.6
Rb^+	*CO_2^- + *Rb(H_2O)	-1.07	5.44	3.9
	*CO_2^- + *Rb(H_2O)$_2$	-0.78	5.22	2.9
	*CO_2^- + *Rb(H_2O)$_3$	-0.47	5.18	1.6
	*CO_2^- + *Rb(H_2O)$_4$	-0.41	5.21	1.4
	*CO_2^- + *Rb(H_2O)$_5$	-0.36	5.30	1.3
Cs^+	*CO_2^- + *Cs(H_2O)	-0.52	5.11	1.5
	*CO_2^- + *Li(H_2O)$_2$	-0.79	5.12	2.2
	*CO_2^- + *Cs(H_2O)$_3$	-0.42	5.21	1.3
	*CO_2^- + *Cs(H_2O)$_4$	-0.38	5.29	1.1
	*CO_2^- + *Cs(H_2O)$_5$	-0.39	5.37	1.2

The predicted EDL widths from the co-adsorbed model suggests that the cation may be close enough to coordinate with $*CO_2^-$ on Au (111) surface. Table **S8** shows that the coordinated cation model is consistent with the experimentally derived symmetry factors.

Table S8. **EDL widths predicted from cation coordinated $*CO_2^-$ reaction path models at $\beta_{DFT} = \beta_{exp}$.** Dipole moment and polarizability of $* CO_2^-$ and *(X)(H2O)n coordinated state on Au (111) surface for X= Li^+, Na^+, Rb^+ and Cs^+. Dipole moment and polarizability changes are relative to the bare metal surface.

This table shows that cation coordinated $* CO_2^-$ models across alkali metal cations show sufficient dipole moment changes and reasonable predicted Helmholtz EDL widths.

Cation	Transition State	Dipole Moment (e^-Å)	Polarizability ($e^-Å^2V^{-1}$)	EDL Width at $\beta_{DFT} = \beta_{exp}$ (Å)
Li^+	$*CO_2Li$	-0.97	4.89	2.9
	$*CO_2Li(H_2O)$	-0.81	5.04	2.6
Na^+	$*CO_2Na$	-1.35	5.08	4.4
	$*CO_2Na(H_2O)$	-1.58	7.38	5.2
Rb^+	$*CO_2Rb$	-1.67	5.42	6.2
	$*CO_2Rb(H_2O)$	-1.66	7.54	6.2
Cs^+	$*CO_2Cs$	-1.73	5.32	5.3
	$*CO_2Cs(H_2O)$	-1.82	7.94	5.6

Supplementary Note 6. Cation and Au Surface Facet Trends

Fig. 7 shows that DFT predicted symmetry factors does not agree with both the surface facet and cation trends given the Helmholtz width is kept constant across all electrolyte composition and surface facets of Au. **Fig. S28-S30** investigates the origin of this disagreement by plotting the symmetry factor obtained from DFT and experiment with the DFT obtained dipole moment change.

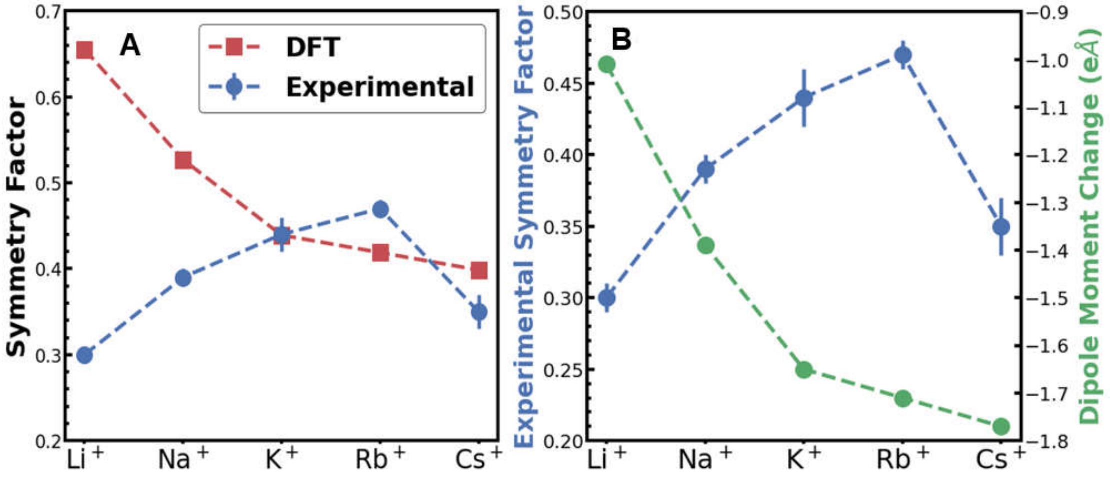

Fig. S28. Comparison of experimental and DFT predicted symmetry factors on Au(111). (A) Comparison of experimental and DFT predicted symmetry factors assuming the EDL width is constant across alkali metal cations on the Au (111) surface. A Helmholtz width of 5.9 Å was used as fitted for $*KCO_2$ to match with β_{exp} on the Au (111) surface. A cation coordinated model represents the transition state for $*CO_2^-$. Uncertainty of β_{exp} denoted based on Figure 5. The dielectric constant of one was used for DFT calculated values of β. **(B)** Experimentally derived symmetry factors and DFT predicted dipole moment changes across different cations on the Au (111) surface. A cation coordinated $*CO_2^-$ model of the transition state is used. Uncertainty values are depicted for experimental values. Dipole moment changes are relative to the bare Au (111) surface.

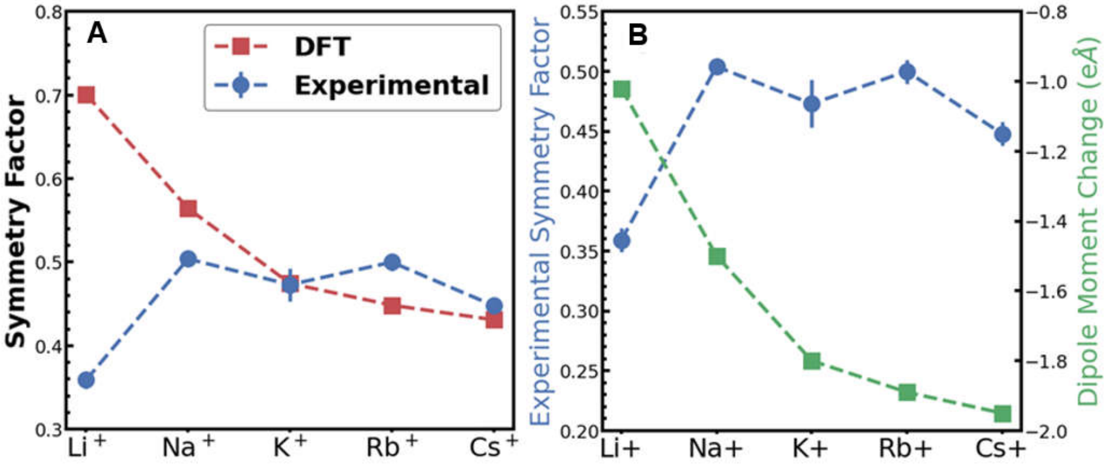

Fig. S29. Comparison of experimental and DFT predicted symmetry factors on Au(110). (A) Comparison of experimental and DFT predicted symmetry factors assuming the EDL width is constant across alkali metal cations on Au (110) surface. A Helmholtz width of 6.9 Å was used as fitted for $*KCO_2$ to match with β_{exp} on Au (110) surface. A cation coordinated model represents the transition state for $*CO_2^-$ on Au (110) surface. The dielectric constant of one was used for DFT calculated values of β. **(B)** Experimentally derived symmetry factors and DFT predicted dipole moment changes across different cations on Au (110) surface. A cation coordinated $*CO_2^-$ model of the transition state is used. Uncertainty values are depicted for experimental values.

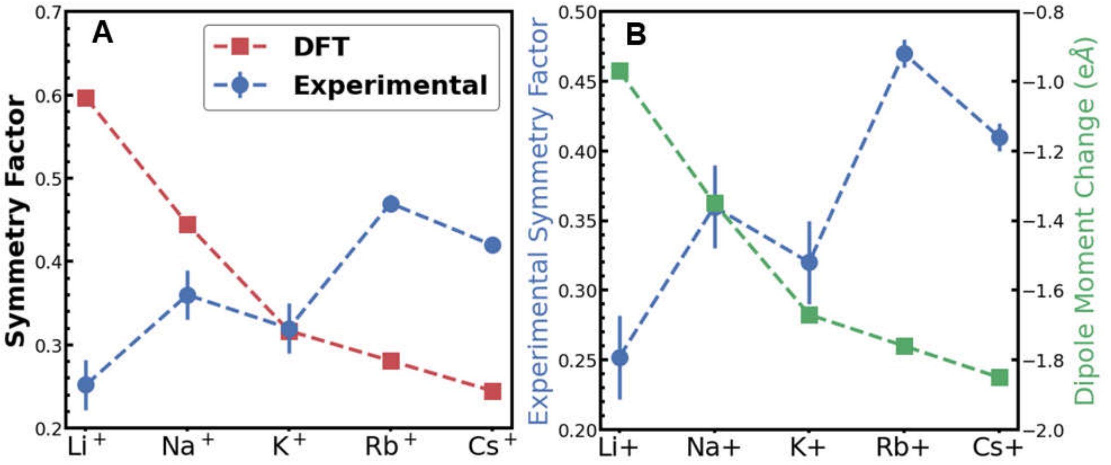

Fig. S30. Comparison of experimental and DFT predicted symmetry factors on Au(100). (A) Comparison of experimental and DFT predicted symmetry factors assuming the EDL width is constant across alkali metal cations on Au (100) surface. A Helmholtz width of 5.0 Å was used as fitted for $^*KCO_2$ to match with β_{exp} on Au (100) surface. A cation coordinated model represents the transition state for $^*CO_2^-$ on Au (100) surface. The dielectric constant of one was used for DFT calculated values of β. **(B)** Experimentally derived symmetry factors and DFT predicted dipole moment changes across different cations on Au (100) surface. A cation coordinated $^*CO_2^-$ model of the transition state is used. Uncertainty values are depicted for experimental values.

Fig. S28 shows that β_{DFT} qualitatively follows the trend of dipole moment change on Au (111) surface. This is not surprising as the Helmholtz EDL width is kept constant (5.9 Å) across differing electrolyte composition changes, resulting in the dipole moment dictating the qualitative trend of β_{DFT}. Comparison between trend of dipole moment changes and β_{exp} clearly shows that the magnitude of the dipole moment changes cannot explain the cation trends on Au (111) surface. **Fig. S29** and **S30** show similar conclusions on the Au (100) and Au (110) surface.

Fig. S31 and Table **S9** shows the trends and values of the dipole moment change of cation assisted $^*CO_2^-$ formation across different surface facets of Au and cation identity.

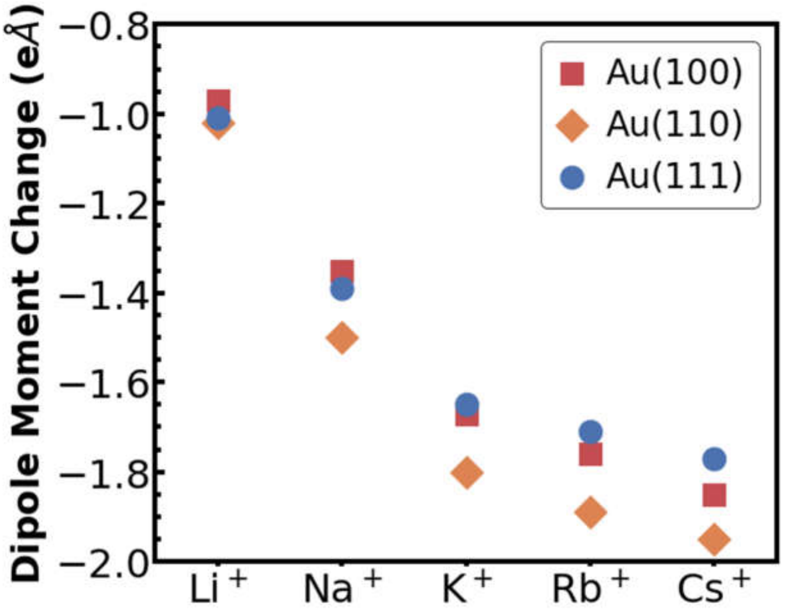

Fig. S31. Dipole moment changes of adsorbed coordinated cation $^*CO_2^-$ complex across different surface facets of Au. Dipole moment changes are relative to the respective bare Au surface facets.

Table S9. Dipole moment of adsorbed coordinated cation $^*CO_2^-$ complex across different surface facets of Au.

**Dipole Moment** (e^- Å)			
**Coordinate Cation-$^*CO_2^-$**	**Au (111)**	**Au (110)**	**Au (100)**
**Li^+**	-0.97	-1.02	-0.97
**Na^+**	-1.35	-1.49	-1.35
**K^+**	-1.67	-1.80	-1.61
**Rb^+**	-1.76	-1.89	-1.67
**Cs^+**	-1.85	-1.95	-1.73
**Bare**	0.01	0.00	0.04

Fig. 7 assumes the EDL width is the same across all cations and surface facets of Au. **Fig. S30** adjusts the EDL width based on each surface facet, where the trends across surface facets would match for when $\beta_{DFT} = \beta_{exp}$ for K^+ containing electrolyte. For the same surface facet, this EDL width is kept constant across all cations.

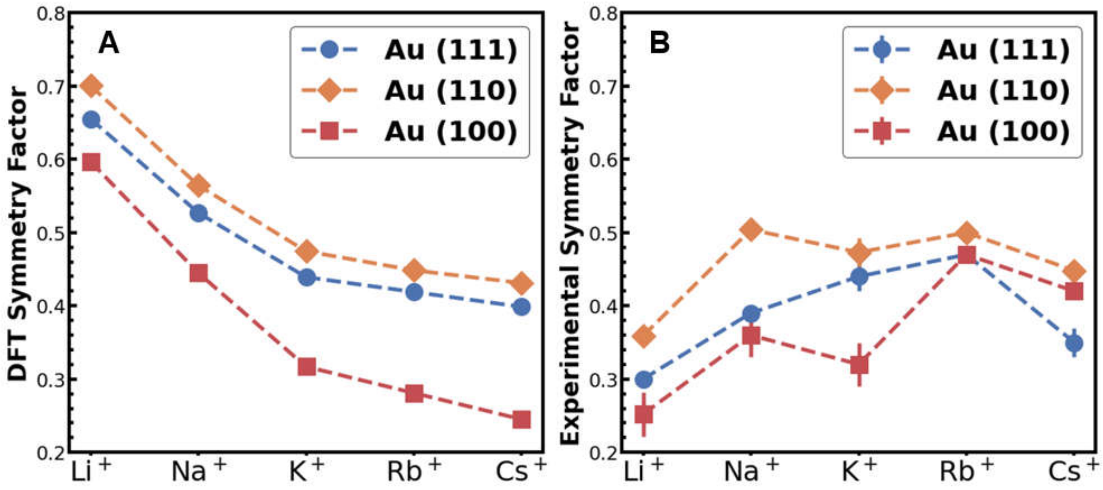

Fig. S32. Comparison of experimental and DFT predicted symmetry factors on Au(hkl). (A) DFT predicted symmetry factors across different alkali metal cations and surface facets of Au. The EDL width is assumed constant across alkali metal cations on the same surface facet. The Helmholtz width for each surface facet of Au was fitted for where $\beta_{DFT} = \beta_{exp}$ for K^+. These EDL widths are respectively 5.9 Å, 6.9 Å, and 5 Å on the Au (111), Au (110), and Au (100) surface. A cation coordinated model represents the transition state for $*CO_2^-$ on Au (111) surface. **(B)** Experimentally derived symmetry factors across different alkali metal cations and surface facets of Au derived from Figure 5. Uncertainty of β_{exp} denoted based on Figure 5.

Fig. S32 shows that ,while the disagreement across cations on the same surface is still evident between DFT and experiment, there is general agreement between symmetry factors across different surface facets for the same cation. For the same cation composition, the symmetry factor increases from Au (100) < Au (111) < Au (110) with

the exception of the Cs^+ composed electrolyte. This predicts that if the classical Helmholtz model upholds that the countercharge distribution must be different across surface facets.

Fig. S33-S35 all consider tuning the EDL width for each cation and surface facet to match $\beta_{DFT} = \beta_{exp}$ based on the respective dipole moment and polarizability change from DFT. Unsurprisingly, $\beta_{DFT} = \beta_{exp}$ once the EDL width is tuned, but DFT alone cannot verify the validity of trends of the EDL widths across alkali metal cations and surface facets due to its inherent limitation in sampling the properties of the electrode-electrolyte interface, relying on methods outside the regime of theory such as Classical MD (*42, 43*). While the exact value of these EDL widths cannot be verified alone from DFT, this suggests that the assumption of the EDL width being constant across different surface facets and electrolyte composition is not necessarily true.

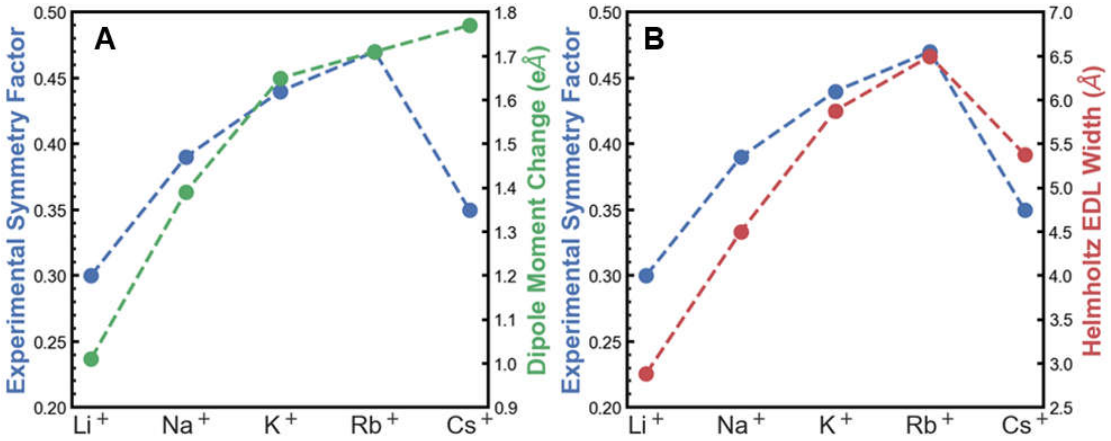

Fig. S33. Comparison of experimental symmetry factors and DFT predicted dipole moment changes on Au (111). (A) Comparison of experimental symmetry factors and DFT predicted dipole moment changes on Au (111) surface. A cation coordinated model represents the transition state for $*CO_2^-$ on Au (111) surface. The dielectric constant of one was used for DFT calculated values of β. Dipole moment changes are relative to the bare metal surface. **(B)** Experimentally derived symmetry factors and predicted EDL widths across different cations on Au (111) surface. EDL widths are fitted for

when $\beta_{DFT} = \beta_{exp}$ for the respective alkali metal cation.

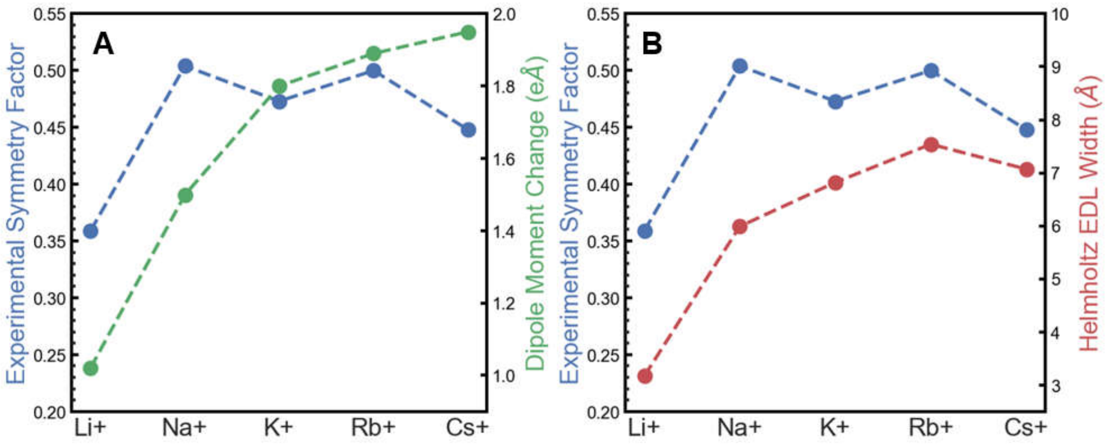

Fig. S34. Comparison of experimental symmetry factors and DFT predicted dipole moment changes on Au (110). (A) Comparison of experimental symmetry factors and DFT predicted dipole moment changes on Au (110) surface. A cation coordinated model represents the transition state for $*CO_2^-$ on Au (110) surface. The dielectric constant of one was used for DFT calculated values of β. Dipole moment changes are relative to the bare metal surface. **(B)** Experimentally derived symmetry factors and predicted EDL widths across different cations on Au (110) surface. EDL widths are fitted for when $\beta_{DFT} = \beta_{exp}$ for the respective alkali metal cation.

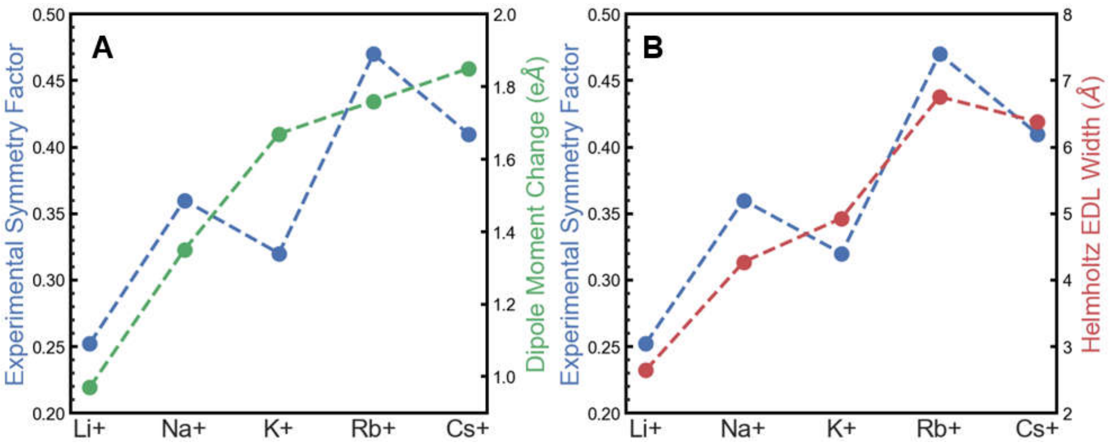

Fig. S35. Comparison of experimental symmetry factors and DFT predicted dipole moment changes on Au (100). (A) Comparison of experimental symmetry factors and DFT predicted dipole moment changes on Au (100) surface. A cation coordinated

model represents the transition state for $*CO_2^-$ on Au (100) surface. The dielectric constant of one was used for DFT calculated values of β. Dipole moment changes are relative to the bare metal surface. **(B)** Experimentally derived symmetry factors and predicted EDL widths across different cations on Au (100) surface. EDL widths are fitted for when $\beta_{DFT} = \beta_{exp}$ for the respective alkali metal cation.

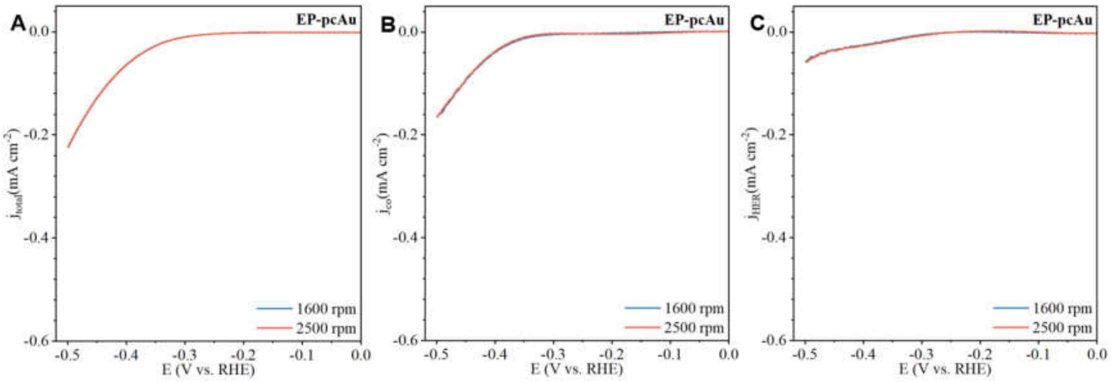

Fig. S36. Rotation-rate dependence experiments. (A) Total current densities, **(B)** CO partial current densities and **(C)** HER current densities obtained in 0.1 M $NaHCO_3$ at different rotation rates.

Table S10. **Determination of the exchange current densities of CO_2R to CO.** The values of exchange current densities fitted from modified Tafel plots of CO_2R on EP-pcAu in 0.1 M bicarbonate electrolytes.

log (j_{co} / mA cm^{-2})	$LiHCO_3$	$NaHCO_3$	$KHCO_3$	$RbHCO_3$	$CsHCO_3$
EP-pcAu	-3.56±0.09	-3.42±0.05	-3.40±0.08	-3.37±0.12	-3.28±0.06

Table S11. Inductively coupled plasma mass spectrometry (ICP-MS) results. The electrolyte concentration is 0.05 M (All the data was taken on a Perkin Elmer Elan DRCII Inductively Coupled Plasma Mass Spectrometer, results are typically accurate to within 10% relative concentration).

Electrolytes	Ni (ppb)	Pb (ppb)	Fe (ppb)
Li_2CO_3	0.2	0.4	5.7
Purified Li_2CO_3	0.3	0.3	< 0.1
Na_2CO_3	0.2	0.4	3.6
Purified Na_2CO_3	0.3	0.2	0.6
K_2CO_3	0.3	0.9	2.0
Purified K_2CO_3	0.3	0.2	< 0.1
Rb_2CO_3	0.6	0.3	4.8
Purified Rb_2CO_3	0.2	< 0.1	0.7
Cs_2CO_3	0.6	0.3	2.6
Purified Cs_2CO_3	0.2	0.1	0.2

REFERENCES AND NOTES

1. S. Nitopi, E. Bertheussen, S. B. Scott, X. Liu, A. K. Engstfeld, S. Horch, B. Seger, I. E. L. Stephens, K. Chan, C. Hahn, J. K. Nørskov, T. F. Jaramillo, I. Chorkendorff, Progress and perspectives of electrochemical CO_2 reduction on copper in aqueous electrolyte. *Chem. Rev.* **119**, 7610–7672 (2019).

2. J.-P. Jones, G. K. S. Prakash, G. A. Olah, Electrochemical CO_2 reduction: Recent advances and current trends. *Isr. J. Chem.* **54**, 1451–1466 (2014).

3. I. E. L. Stephens, K. Chan, A. Bagger, S. W. Boettcher, J. Bonin, E. Boutin, A. K. Buckley, R. Buonsanti, E. R. Cave, X. Chang, S. W. Chee, A. H. M. da Silva, P. de Luna, O. Einsle, B. Endrődi, M. Escudero-Escribano, J. V. Ferreira de Araujo, M. C. Figueiredo, C. Hahn, K. U. Hansen, S. Haussener, S. Hunegnaw, Z. Huo, Y. J. Hwang, C. Janáky, B. S. Jayathilake, F. Jiao, Z. P. Jovanov, P. Karimi, M. T. M. Koper, K. P. Kuhl, W. H. Lee, Z. Liang, X. Liu, S. Ma, M. Ma, H.-S. Oh, M. Robert, B. R. Cuenya, J. Rossmeisl, C. Roy, M. P. Ryan, E. H. Sargent, P. Sebastián-Pascual, B. Seger, L. Steier, P. Strasser, A. S. Varela, R. E. Vos, X. Wang, B. Xu, H. Yadegari, Y. Zhou, 2022 roadmap on low temperature electrochemical CO_2 reduction. *J. Phys. Energy* **4**, 042003 (2022).

4. M. Akira, H. Yoshio, Product selectivity affected by cationic species in electrochemical reduction of CO_2 and CO at a Cu electrode. *Bull. Chem. Soc. Jpn.* **64**, 123–127 (1991).

5. S. Ringe, E. L. Clark, J. Resasco, A. Walton, B. Seger, A. T. Bell, K. Chan, Understanding cation effects in electrochemical CO_2 reduction. *Energy Environ. Sci.* **12**, 3001–3014 (2019).

6. M. R. Thorson, K. I. Siil, P. J. A. Kenis, Effect of cations on the electrochemical conversion of CO_2 to CO. *J. Electrochem. Soc.* **160**, F69–F74 (2013).

7. J. Resasco, L. D. Chen, E. Clark, C. Tsai, C. Hahn, T. F. Jaramillo, K. Chan, A. T. Bell, Promoter effects of alkali metal cations on the electrochemical reduction of carbon dioxide. *J. Am. Chem. Soc.* **139**, 11277–11287 (2017).

8. O. Ayemoba, A. Cuesta, Spectroscopic evidence of size-dependent buffering of interfacial pH by cation hydrolysis during CO_2 electroreduction. *ACS Appl. Mater. Interfaces* **9**, 27377–27382 (2017).

9. M. R. Singh, Y. Kwon, Y. Lum, J. W. Ager, III, A. T. Bell, Hydrolysis of electrolyte cations enhances the electrochemical reduction of CO_2 over Ag and Cu. *J. Am. Chem. Soc.* **138**, 13006–13012 (2016).

10. G. Hussain, L. Pérez-Martínez, J.-B. Le, M. Papasizza, G. Cabello, J. Cheng, A. Cuesta, How cations determine the interfacial potential profile: Relevance for the CO_2 reduction reaction. *Electrochim. Acta* **327**, 135055 (2019).

11. M. C. O. Monteiro, F. Dattila, B. Hagedoorn, R. García-Muelas, N. López, M. T. M. Koper, Absence of CO_2 electroreduction on copper, gold and silver electrodes without metal cations in solution. *Nat. Catal.* **4**, 654–662 (2021).

12. V. J. Ovalle, Y.-S. Hsu, N. Agrawal, M. J. Janik, M. M. Waegele, Correlating hydration free energy and specific adsorption of alkali metal cations during CO_2 electroreduction on Au. *Nat. Catal.* **5**, 624–632 (2022).

13. X. Chen, I. T. McCrum, K. A. Schwarz, M. J. Janik, M. T. M. Koper, Co-adsorption of cations as the cause of the apparent pH dependence of hydrogen adsorption on a stepped platinum single-crystal electrode. *Angew. Chem. Int. Ed.* **56**, 15025–15029 (2017).

14. Z.-Q. Zhang, S. Banerjee, V. S. Thoi, A. Shoji Hall, Reorganization of interfacial water by an amphiphilic cationic surfactant promotes CO_2 reduction. *J. Phys. Chem. Lett.* **11**, 5457–5463 (2020).

15. J. Li, X. Li, C. M. Gunathunge, M. M. Waegele, Hydrogen bonding steers the product selectivity of electrocatalytic CO reduction. *Proc. Natl. Acad. Sci. U.S.A.* **116**, 9220–9229 (2019).

16. A. S. Malkani, J. Li, N. J. Oliveira, M. He, X. Chang, B. Xu, Q. Lu, Understanding the electric and nonelectric field components of the cation effect on the electrochemical CO reduction reaction. *Sci. Adv.* **6**, eabd2569 (2020).

17. Q. Zhu, S. K. Wallentine, G.-H. Deng, J. A. Rebstock, L. R. Baker, The solvation-induced onsager reaction field rather than the double-layer field controls CO_2 reduction on gold. *JACS Au* **2**, 472–482 (2022).

18. S.-J. Shin, H. Choi, S. Ringe, D. H. Won, H.-S. Oh, D. H. Kim, T. Lee, D.-H. Nam, H. Kim, C. H. Choi, A unifying mechanism for cation effect modulating C1 and C2 productions from CO_2 electroreduction. *Nat. Commun.* **13**, 5482 (2022).

19. X. Qin, T. Vegge, H. A. Hansen, Cation-coordinated inner-sphere CO_2 electroreduction at Au–water interfaces. *J. Am. Chem. Soc.* **145**, 1897–1905 (2023).

20. M. M. Waegele, C. M. Gunathunge, J. Li, X. Li, How cations affect the electric double layer and the rates and selectivity of electrocatalytic processes. *J. Chem. Phys.* **151**, 160902 (2019).

21. A. S. Malkani, J. Anibal, B. Xu, Cation effect on interfacial CO_2 concentration in the electrochemical CO_2 reduction reaction. *ACS Catalysis* **10**, 14871–14876 (2020).

22. E. Pérez-Gallent, G. Marcandalli, M. C. Figueiredo, F. Calle-Vallejo, M. T. M. Koper, Structure- and potential-dependent cation effects on CO reduction at copper single-crystal electrodes. *J. Am. Chem. Soc.* **139**, 16412–16419 (2017).

23. B. A. Zhang, T. Ozel, J. S. Elias, C. Costentin, D. G. Nocera, Interplay of homogeneous reactions, mass transport, and kinetics in determining selectivity of the reduction of CO_2 on gold electrodes. *ACS Cent. Sci.* **5**, 1097–1105 (2019).

24. K. R. M. Corpus, J. C. Bui, A. M. Limaye, L. M. Pant, K. Manthiram, A. Z. Weber, A. T. Bell, Coupling covariance matrix adaptation with continuum modeling for determination of kinetic parameters associated with electrochemical CO_2 reduction. *Joule* **7**, 1289–1307 (2023).

25. A. Goyal, G. Marcandalli, V. A. Mints, M. T. M. Koper, Competition between CO_2 reduction and hydrogen evolution on a gold electrode under well-defined mass transport conditions. *J. Am. Chem. Soc.* **142**, 4154–4161 (2020).

26. Z. Cui, A. J.-W. Wong, M. J. Janik, A. C. Co, Negative reaction order for CO during CO_2 electroreduction on Au. *J. Am. Chem. Soc.* **146**, 23872–23880 (2024).

27. A. Hamelin, Cyclic voltammetry at gold single-crystal surfaces. Part 1. Behaviour at low-index faces. *J. Electroanal. Chem.* **407**, 1–11 (1996).

28. A. Dakkouri, D. Kolb, *Reconstruction of Gold Surfaces* (Marcel Dekker, 1999).

29. C. Jeyabharathi, P. Ahrens, U. Hasse, F. Scholz, Identification of low-index crystal planes of polycrystalline gold on the basis of electrochemical oxide layer formation. *J. Solid State Electrochem.* **20**, 3025–3031 (2016).

30. S.-G. Sun, W.-B. Cai, L.-J. Wan, M. Osawa, Infrared absorption enhancement for CO adsorbed on Au films in perchloric acid solutions and effects of surface structure studied by cyclic voltammetry, scanning tunneling microscopy, and surface-enhanced IR spectroscopy. *J. Phys. Chem. B.* **103**, 2460–2466 (1999).

31. S. Štrbac, R. R. Adžć, A. Hamelin, Oxide formation on gold single crystal stepped surfaces. *J. Electroanal. Chem. Interf. Electrochem.* **249**, 291–310 (1988).

32. J. A. Rebstock, Q. Zhu, L. R. Baker, Exploring the influence of interfacial solvation on electrochemical CO_2 reduction using plasmon-enhanced vibrational sum frequency generation spectroscopy. *ChemCatChem* **16**, e202301301 (2024).

33. Q. Dong, X. Zhang, D. He, C. Lang, D. Wang, Role of H_2O in CO_2 electrochemical reduction as studied in a water-in-salt system. *ACS Cent. Sci.* **5**, 1461–1467 (2019).

34. S. Ringe, C. G. Morales-Guio, L. D. Chen, M. Fields, T. F. Jaramillo, C. Hahn, K. Chan, Double layer charging driven carbon dioxide adsorption limits the rate of electrochemical carbon dioxide reduction on Gold. *Nat. Commun.* **11**, 33 (2020).

35. W. Deng, P. Zhang, B. Seger, J. Gong, Unraveling the rate-limiting step of two-electron transfer electrochemical reduction of carbon dioxide. *Nat. Commun.* **13**, 803 (2022).

36. S. Mezzavilla, S. Horch, I. E. L. Stephens, B. Seger, I. Chorkendorff, Structure sensitivity in the electrocatalytic reduction of CO_2 with gold catalysts. *Angew. Chem. Int. Ed.* **58**, 3774–3778 (2019).

37. S. A. Akhade, N. J. Bernstein, M. R. Esopi, M. J. Regula, M. J. Janik, A simple method to approximate electrode potential-dependent activation energies using density functional theory. *Catal. Today* **288**, 63–73 (2017).

38. X. Nie, M. R. Esopi, M. J. Janik, A. Asthagiri, Selectivity of CO_2 reduction on copper electrodes: The role of the kinetics of elementary steps. *Angew. Chem. Int. Ed.* **52**, 2459–2462 (2013).

39. N. Agrawal, A. J.-W. Wong, S. Maheshwari, M. J. Janik, An efficient approach to compartmentalize double layer effects on kinetics of interfacial proton-electron transfer reactions. *J. Catal.* **430**, 115360 (2024).

40. L. Fumagalli, A. Esfandiar, R. Fabregas, S. Hu, P. Ares, A. Janardanan, Q. Yang, B. Radha, T. Taniguchi, K. Watanabe, G. Gomila, K. S. Novoselov, A. K. Geim, Anomalously low dielectric constant of confined water. *Science* **360**, 1339–1342 (2018).

41. B. Tran, Y. Zhou, M. J. Janik, S. T. Milner, Negative dielectric constant of water at a metal interface. *Phys. Rev. Lett.* **131**, 248001 (2023).

42. R. Sundararaman, D. Vigil-Fowler, K. Schwarz, Improving the accuracy of atomistic simulations of the electrochemical interface. *Chem. Rev.* **122**, 10651–10674 (2022).

43. K. Schwarz, R. Sundararaman, The electrochemical interface in first-principles calculations. *Surf. Sci. Rep.* **75**, 100492 (2020).

44. A. Shandilya, K. Schwarz, R. Sundararaman, Interfacial water asymmetry at ideal electrochemical interfaces. *J. Chem. Phys.* **156**, 014705 (2022).

45. J. J. Karnes, S. E. Weitzner, S. A. Akhade, S. E. Baker, E. B. Duoss, J. B. Varley, A hybrid quantum–classical study of ion adsorption at the copper electrode. *J. Phys. Chem. C* **126**, 12413–12423 (2022).

46. K.-Y. Yeh, M. J. Janik, in *Computational Catalysis*, A. Asthagiri, M. J. Janik, Eds. (The Royal Society of Chemistry, 2013), pp. 116–156.

47. S. Maheshwari, Y. Li, N. Agrawal, M. J. Janik, in *Advances in Catalysis*, C. Song, Ed. (Academic Press, 2018), vol. 63, pp. 117–167.

48. A. J.-W. Wong, B. Tran, N. Agrawal, B. R. Goldsmith, M. J. Janik, Sensitivity analysis of electrochemical double layer approximations on electrokinetic predictions: Case study for CO reduction on copper. *J. Phys. Chem. C* **128**, 10837–10847 (2024).

49. Z. Cui, M. A. Marx, M. N. Tegomoh, A. C. Co, A guide to evaluate electrolyte purity for CO_2 reduction studies. *ACS Energy Lett.* **8**, 5201–5205 (2023).

50. Y. Yang, R. G. Agarwal, P. Hutchison, R. Rizo, A. V. Soudackov, X. Lu, E. Herrero, J. M. Feliu, S. Hammes-Schiffer, J. M. Mayer, H. D. Abruña, Inverse kinetic isotope effects in the oxygen reduction reaction at platinum single crystals. *Nat. Chem.* **15**, 271–277 (2023).

51. D. A. J. Rand, R. Woods, The nature of adsorbed oxygen on rhodium, palladium and gold electrodes. *J. Electroanal. Chem. Interf. Electrochem.* **31**, 29–38 (1971).

52. G. Kresse, J. Hafner, Ab initio molecular dynamics for liquid metals. *Phys. Rev. B* **47**, 558–561 (1993).

53. G. Kresse, J. Hafner, Ab initio molecular-dynamics simulation of the liquid-metal–amorphous-semiconductor transition in germanium. *Phys. Rev. B* **49**, 14251–14269 (1994).

54. G. Kresse, J. Furthmüller, Efficiency of ab-initio total energy calculations for metals and semiconductors using a plane-wave basis set. *Comput. Mater. Sci.* **6**, 15–50 (1996).

55. G. Kresse, J. Furthmüller, Efficient iterative schemes for ab initio total-energy calculations using a plane-wave basis set. *Phys Rev. B* **54**, 11169–11186 (1996).

56. G. Kresse, D. Joubert, From ultrasoft pseudopotentials to the projector augmented-wave method. *Phys. Rev. B* **59**, 1758–1775 (1999).

57. P. E. Blöchl, Projector augmented-wave method. *Phys. Rev. B* **50**, 17953–17979 (1994).

58. J. P. Perdew, K. Burke, M. Ernzerhof, Generalized gradient approximation made simple. *Phys. Rev. Lett.* **77**, 3865–3868 (1996).

59. J. P. Perdew, K. Burke, M. Ernzerhof, Generalized gradient approximation made simple [Phys. Rev. Lett. 77, 3865 (1996)]. *Phys. Rev. Lett.* **78**, 1396–1396 (1997).

60. J. P. Perdew, J. A. Chevary, S. H. Vosko, K. A. Jackson, M. R. Pederson, D. J. Singh, C. Fiolhais, Atoms, molecules, solids, and surfaces: Applications of the generalized gradient approximation for exchange and correlation. *Phys. Rev. B* **46**, 6671–6687 (1992).

61. M. Methfessel, A. T. Paxton, High-precision sampling for Brillouin-zone integration in metals. *Phys. Rev. B* **40**, 3616–3621 (1989).

62. H. J. Monkhorst, J. D. Pack, Special points for Brillouin-zone integrations. *Phys. Rev. B* **13**, 5188–5192 (1976).

63. J. D. Pack, H. J. Monkhorst, "Special points for Brillouin-zone integrations"—A reply. *Phys. Rev. B* **16**, 1748–1749 (1977).

64. N. Devi, C. K. Williams, A. Chaturvedi, J. J. Jiang, Homogeneous electrocatalytic CO_2 reduction using a porphyrin complex with flexible triazole units in the second coordination sphere. *ACS Appl. Energy Mater.* **4**, 3604–3611 (2021).

65. N. Todoroki, H. Tei, H. Tsurumaki, T. Miyakawa, T. Inoue, T. Wadayama, Surface atomic arrangement dependence of electrochemical CO_2 reduction on gold: Online electrochemical mass spectrometric study on low-index Au(hkl) surfaces. *ACS Catal.* **9**, 1383–1388 (2019).

66. G. Marcandalli, M. Villalba, M. T. M. Koper, The importance of acid–base equilibria in bicarbonate electrolytes for CO_2 electrochemical reduction and CO reoxidation studied on Au(hkl) electrodes. *Langmuir* **37**, 5707–5716 (2021).

67. A. Wuttig, M. Yaguchi, K. Motobayashi, M. Osawa, Y. Surendranath, Inhibited proton transfer enhances Au-catalyzed CO_2-to-fuels selectivity. *Proc. Natl. Acad. Sci. U.S.A.* **113**, E4585-E4593 (2016).

68. Y. Hori, in *Modern Aspects of Electrochemistry*, C. G. Vayenas, R. E. White, M. E. Gamboa-Aldeco, Eds. (Springer New York, 2008), pp. 89–189.

69. M. Dunwell, W. Luc, Y. Yan, F. Jiao, B. Xu, Understanding surface-mediated electrochemical reactions: CO_2 reduction and beyond. *ACS Catal.* **8**, 8121–8129 (2018).

70. G. Rostamikia, M. J. Janik, Borohydride oxidation over Au(111): A first-principles mechanistic study relevant to direct borohydride fuel cells. *J. Electrochem. Soc.* **156**, B86–B92 (2009).

71. H. A. Schwarz, R. W. Dodson, Reduction potentials of CO_2- and the alcohol radicals. *J. Phys. Chem.* **93**, 409–414 (1989).
